# Probing Proximal Intramolecular Hydrogen Bonding Interactions on a Norbornane Scaffold

**DOI:** 10.1021/acs.jpca.5c08010

**Published:** 2026-01-28

**Authors:** Carly A. Rock, Dakota B. Green, Martin J. Flores, Jeremy M. Carr, Thufail M. Ismail, Gregory S. Tschumper

**Affiliations:** † Oxford High School, Oxford, Mississippi 38655, United States; ‡ 31632 Central Alabama Community College, Alexander City, Alabama 35010, United States; § Department of Chemistry, 14717 Missouri University of Science and Technology, Rolla, Missouri 65409, United States

## Abstract

A systematic computational study was conducted on 12 unique 2,6-disubstituted norbornane derivatives to assess the ability of various acceptor groups (A = F, Cl, Br, OH, OCH_3_, SH, SCH_3_, NHCH_3_, N­(CH_3_)_2_, PH_2_, PHCH_3_, and P­(CH_3_)_2_) to engage in intramolecular hydrogen bonding with a proximal OH donor. 32 unique minima have been characterized with M06-2X and df-MP2 geometry optimizations as well as PNO-LCCSD­(T)-F12 single point energy computations. Conformations with the OH donor oriented toward the acceptor to form an OH···A contact (+HB) consistently had lower electronic energies (by approximately 2 to 8 kcal mol^–1^) than their counterparts with the OH directed away from the acceptor (−HB). Additional M06-2X computations revealed that these intramolecular contacts also induce significant shifts to lower energy in the OH stretching frequencies (usually by −50 to −100 cm^–1^, but more than −200 cm^–1^ for OH···N interactions) and notable deshielding of the hydrogen atom (with shifts in the isotropic nuclear magnetic resonance (NMR) chemical shielding constants ranging from −1 to −5 ppm). Quantum theory of atoms in molecules (QTAIM) analysis confirms the presence of bond critical points with electron densities (ca. 0.02 to 0.03 *e* bohr^–3^) that are within the typical range of values for hydrogen bonds. These findings demonstrate the capacity of not only N and O, but also P and S, acceptor groups, to establish appreciable attractive intramolecular interactions with a proximal OH group on a rather rigid molecular scaffold.

## Graphical abstract

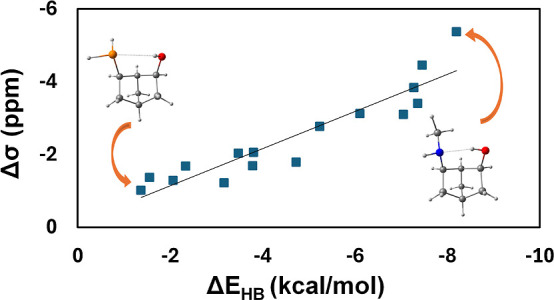

## 1 Introduction

It is well-known that hydrogen bonding plays a critical role in numerous biological, chemical, and physical processes.^1–10^ Although intermolecular and intramolecular variations of hydrogen bonding occur, there are some fundamental differences between the two phenomena, many of which were reviewed by Nagy in 2014.^11^ Their computational and theoretical characterizations also differs. In the case of intermolecular hydrogen bonding, characterizing the interaction between a hydrogen bond donor and acceptor on separate molecules (generally represented as X–H···A–R, where X–H is the hydrogen bond donor and A–R is the acceptor) is fairly straightforward. Within the supermolecular approach, for example, the energy of the intermolecular hydrogen bond (*E*_HB_) can be calculated by subtracting the energy of the individual hydrogen bond donor (X–H) and acceptor (A–R) fragments from the total energy of the hydrogen-bonded complex (X–H···A–R).^12,13^ In contrast, estimating the energy of an intramolecular hydrogen bond (where the donor and acceptor groups are located on the same molecule) presents a challenge due to the difficulty of isolating an interaction between two functional groups in the same molecule.

Scheiner nicely summarized^14^ this technical obstacle that had been recognized since some of the earliest theoretical studies of intramolecular hydrogen bonding.^15^ Over the years, a number of theoretical investigations have employed a range of different procedures (or “tricks”^14^) to estimate the strengths of intramolecular interactions in a variety of systems,^16–36^ many of which have been summarized in a fairly recent review by Jabłoński.^26^ A common approach is a straightforward conformational analysis that calculates the energy difference (Δ*E*) between two conformers, one with and one without the intramolecular hydrogen bond (denoted here as +HB and −HB, respectively and depicted in Figure 1 for an OH donor and a generic acceptor group A). Although this method can provide reasonable estimates of the associated intramolecular hydrogen bond strengths,^32^ its results may be influenced by competing energetic contributions. For example, Δ*E* can overestimate the hydrogen bond strength when unfavorable interactions (e.g., lone pair/lone pair repulsion) are present between the hydrogen bond donor and acceptor groups in the −HB conformer by increasing the energy of the reference structure lacking the intramolecular hydrogen bond.^33,34,36^ In contrast, significant structural distortion upon formation of the intramolecular hydrogen bond can yield Δ*E* values that underestimate the strength of the interaction.^37^ Although this conformational energetic analysis can be useful, it is more reliable when combined with the analysis of other properties associated with intramolecular hydrogen bonding (e.g., structural distortions, shifts in spectroscopic signals, and features of the electron densities).^38^

**Figure 1:**
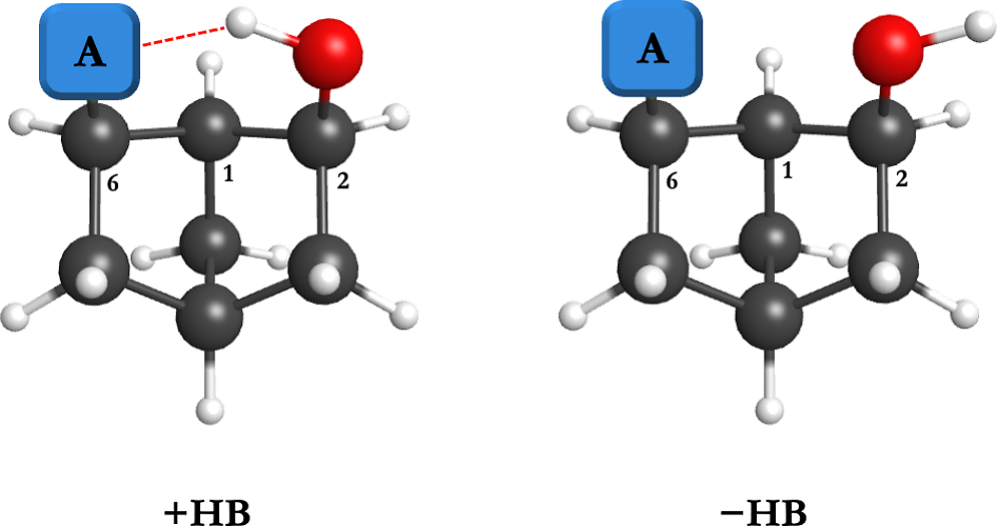
Generic depiction of the 2,6-disubstituted norbornanes with an OH donor and various acceptor groups (represented as “A” here) with (+HB) and without (−HB) an attractive intramolecular contact (A = F, Cl, Br, OH, OCH_3_, SH, SCH_3_, NHCH_3_, N­(CH_3_)_2_, PH_2_, PHCH_3_, and P­(CH_3_)_2_).

This work extends recent studies of intramolecular hydrogen bonding^28,38^ to a more rigid norbornane skeleton (bicyclo[2.2.1]­heptane) in order to reduce potential energetic contributions from geometric distortion of the molecular framework. Norbornanes are structurally recognized as bridged analogs of a cyclohexane boat conformation.^39^ The norbornane structure has been widely employed across multiple subdisciplines of chemical research. The norbornyl scaffold has proven particularly valuable in studies of noncovalent interactions, where conformational restriction enables clear isolation of through-space effects, including intramolecular hydrogen bonding and frustrated Lewis pair behavior.^40–43^ Synthetically, norbornane derivatives continue to serve as versatile platforms for accessing novel and highly constrained polycyclic frameworks, facilitating the construction of complex ring systems that are otherwise difficult to obtain.^44^ Within the realm of medicinal chemistry, the rigid and lipophilic nature of the norbornane motif has led to its presence in drug design,^45^ including applications in antibacterial agents with activity against methicillin-resistant *Staphylococcus aureus* ^46–48^ and nicotinic acetylcholine receptor agonists.^49^ Norbornane also occupies a distinctive niche in modern materials and energy research. Its structural rigidity has been exploited in the design of covalent organic frameworks for gas separation,^50^ molecular container systems,^51^ and amphiphilic materials.^52^ More recently, norbornane-based hydrocarbons have shown promise as high-density fuel components, where compact, strained frameworks afford favorable energetic and performance characteristics.^53^

In order to investigate the ability of different functional groups to establish an attractive intramolecular contact with a proximal hydroxyl donor group on a norbornane scaffold, the norbornane skeleton was functionalized at the C–2 and C–6 positions, as depicted in Figure 1, positioning an OH donor at C–2 and a variety of hydrogen bond acceptor groups at C–6 (A = F, Cl, Br, OH, OCH_3_, SH, SCH_3_, NHCH_3_, N­(CH_3_)_2_, PH_2_, PHCH_3_, and P­(CH_3_)_2_). Following previous work,^28,38^ structures both with (+HB) and without (−HB) an attractive intramolecular contact are characterized by rotating the OH donor toward and away from the various hydrogen bond acceptor groups, respectively.

Although the qualifying features of hydrogen bonding are arguable,^54^ many studies show that intramolecular hydrogen bonding can play a notable role in conformer stabilization,^30,55–62^ with several experimental and theoretical cases providing evidence of intramolecular hydrogen bonding outside of the conventional definition.^54,63–65^ The structural, spectroscopic, and energetic changes induced by these attractive OH···A intramolecular contacts are characterized here for each system, with particular attention to changes in O–H donor bond lengths, the O–H stretching frequencies, NMR chemical shielding constants, and electron densities calculated at bond critical points (BCP) along the OH···A contacts. To simplify the discussion of the relative energetics presented in this work, the term “hydrogen bond” is used to describe any stabilizing OH···A contact also exhibiting spectroscopic perturbations consistent with hydrogen-bonding interactions.

## 2 Computational Details

To probe the ability of various acceptor groups (A = F, Cl, Br, OH, OCH_3_, SH, SCH_3_, NHCH_3_, N­(CH_3_)_2_, PH_2_, PHCH_3_, and P­(CH_3_)_2_) to form an attractive intramolecular contact (or hydrogen bond) with a proximal OH donor group, a conformation with the intramolecular hydrogen bond (+HB) and the analogous conformation without the intramolecular hydrogen bond (−HB) were examined. In every case, the donor OH group was placed on the C-2 atom of norbornane, and the acceptor group was placed on the C-6 atom. Of the possible hydrogen bond donor–acceptor combinations investigated, 16 unique +HB and 16 unique −HB structures were identified. Note that NH_2_ is not included in this set of A substituents at the C-6 position of norbornane because of its propensity to act as a hydrogen bond donor rather than an acceptor with the OH group at C-2, and it will be examined in a subsequent study of other potential intramolecular hydrogen bond donors in this system.

Full geometry optimizations and harmonic vibrational frequency computations were performed on this set of 32 structures using the M06-2X density functional theory method^66^ and the correlation consistent cc-pVTZ (TZ) basis set.^67–69^ A previous study of intramolecular hydrogen bonding in similar systems^38^ showed that results obtained with the cc-pV­(T+d)­Z for P, S, and Cl were essentially indistinguishable from those obtained with the cc-pVTZ basis set. Therefore, we did not use basis sets with an additional set of tight d-functions for those atoms in this investigation. NMR chemical shielding constants were also calculated^70^ at the same level of theory using the gauge-independent atomic orbital method.^71^ An analysis of the electron density was carried out at the M06-2X/TZ level of theory within the QTAIM^54,72,73^ framework for all of the +HB structures. The bond paths and critical points associated with the OH···A contacts were identified with the Multiwfn software^74^ and visualized with VMD.^75^

Although previous literature characterizing similar systems suggests that M06-2X/TZ results should be reliable,^28,38,76,77^ additional optimizations were performed on all 32 structures using the density-fitted MP2 (df-MP2) method^78–81^ with a pair of triple-ζ basis sets (cc-pVTZ (TZ) and aug-cc-pVTZ (aTZ)). PNO-LCCSD­(T)-F12 ^82,83^ single-point energy computations with the cc-pVTZ (TZ) and cc-pVTZ-F12 (TZ-F12) basis sets^84^ were carried out on the df-MP2/TZ optimized structures.

All M06-2X computations were carried out using the default numerical integration grid within Gaussian16,^85^ and all df-MP2 computations were carried out with the default auxiliary basis sets within PSI4.^86,87^ For all geometry optimizations, the max forces were converged to 1.5 × 10^–5^ *E*_h_ au^–1^. All PNO-LCCSD­(T)-F12 computations were carried out using the default auxiliary basis sets and thresholds within MOLPRO.^88^ For the PNO-LCCSD­(T)-F12 computations, the triples contribution to the energy was not scaled, and results obtained using the F12b approximation are presented here.

## 3 Results and Discussion

### 3.1 Energetic Analysis

Following previous work,^28,38^ M06-2X/TZ full geometry optimizations were carried out on the +HB and −HB 2,6-disubstituted norbornane systems depicted in Figure 1, where A = F, Cl, Br, OH, OCH_3_, SH, SCH_3_, NHCH_3_, N­(CH_3_)_2_, PH_2_, PHCH_3_, and P­(CH_3_)_2_. Sixteen unique conformations with an attractive intramolecular contact between the OH donor group and the various hydrogen bond acceptors were identified and are shown in Figure 2. Note that two distinct +HB conformations were identified for the OCH_3_, SCH_3_, NHCH_3_, and PHCH_3_ acceptor groups. The “anti” and “gauche” labels used in Figure 2 correspond to C1–C6-X-C torsional angles (where X = O, S, N, or P) of approximately ±170° and ±60°, respectively. For every unique +HB conformation, a corresponding −HB conformation was identified, in which the OH donor group was oriented away from the hydrogen bond acceptor group. Harmonic vibrational frequency computations confirmed that the 32 structures were all minima on the M06-2X/TZ potential energy surface.

**Figure 2:**
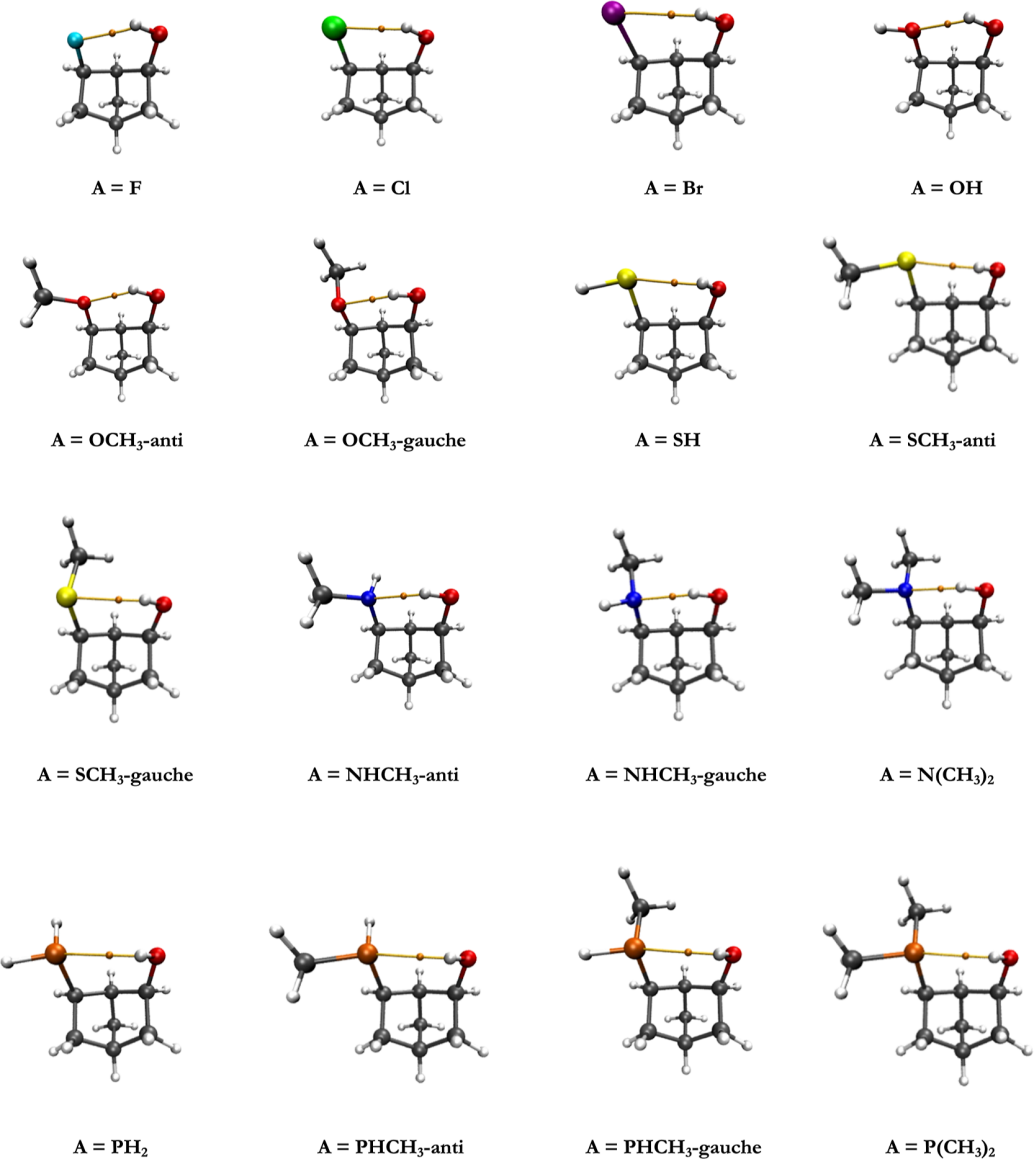
M06-2X/TZ optimized 2,6-disubstituted norbornane +HB conformations with an OH donor and various acceptor groups (A) along with the associated bond critical point (BCP) (orange sphere) along the OH···A contact.

As noted in the introduction, the attractive OH···A contact can be estimated through a conformational analysis by comparing the +HB and −HB electronic energies (*E*_+HB_ and *E*_–HB_) for each substituted norbornane system within this study, in a manner consistent with related work on cyclic carbon scaffolds^28,38^ (Δ*E*_HB_ = *E*_+HB_ −*E*_–HB_). The Δ*E*_HB_ values for the M06-2X/TZ optimized structures are reported in the first column of the numerical data in Table 1. The subsequent two columns contain Δ*E*_HB_ values obtained from the additional df-MP2 optimizations carried out with two triple-ζ basis sets (TZ and aTZ). These are followed by results from PNO-LCCSD­(T)-F12 single-point computations, performed on the df-MP2/TZ optimized structures, using TZ and TZ-F12 basis sets (see the last two columns in Table 1). These calculations were included to assess potential effects from higher-order correlation and basis set incompleteness on intramolecular hydrogen bonding in these norbornane systems. Across all five levels of theory, Δ*E*_HB_ values show that the +HB arrangement exhibiting the OH···A contact consistently has a stabilizing effect on the 2,6-disubstituted norbornane systems and consistently has a lower electronic energy than the −HB structure, ranging in magnitude from around 2.0 kcal mol^–1^ or less for A = SH, SCH_3_ (with anti-conformation), and PH_2_ to more than 8 kcal mol^–1^ for the gauche conformation of the NHCH_3_ acceptor group. Regardless of the level of theory, the most significant degree of stabilization imparted by the OH···A contacts occurs for hydrogen bond acceptor groups containing N and O, resulting in energy differences between the +HB and −HB conformations generally on the order of 7 kcal mol^–1^ or larger.

**Table 1:** Relative Electronic Energies (Δ*E*_HB_ in kcal mol^–1^) Obtained from M06-2X/TZ, df-MP2/TZ, and df-MP2/aTZ Optimizations Followed by PNO-LCCSD­(T)-F12 Single-point Energies Using the TZ and TZ-F12 Basis Sets on the df-MP2/TZ-Optimized Geometries

	M06-2X	df-MP2		PNO-LCCSD(T)-F12	
acceptor	TZ	TZ	aTZ	TZ	TZ-F12
F	–4.73	–4.46	–4.37	–4.54	–4.58
Cl	–3.81	–3.74	–3.75	–3.72	–3.68
Br	–3.48	–3.53	–3.86	–3.38	–3.35
OH	–7.36	–7.15	–6.85	–7.05	–6.83
OCH_3_-anti	–7.05	–7.06	–6.96	–6.90	–6.84
OCH_3_-gauche	–5.24	–5.47	–5.19	–5.36	–5.12
SH	–2.07	–2.16	–2.04	–2.08	–1.99
SCH_3_-anti	–1.56	–1.71	–1.86	–1.62	–1.78
SCH_3_-gauche	–6.11	–6.41	–6.34	–6.19	–6.12
NHCH_3_-anti	–7.28	–7.70	–7.45	–7.60	–7.40
NHCH_3_-gauche	–8.20	–8.71	–8.39	–8.50	–8.15
N(CH_3_)_2_	–7.46	–8.06	–7.75	–7.71	–7.37
PH_2_	–1.37	–1.67	–1.68	–1.60	–1.57
PHCH_3_-anti	–2.34	–2.62	–2.72	–2.51	–2.57
PHCH_3_-gauche	–3.17	–3.42	–3.40	–3.33	–3.32
P(CH_3_)_2_	–3.79	–4.11	–4.12	–4.00	–4.02

Focusing on the PNO-LCCSD­(T)-F12 data computed with the TZ-F12 basis set (last column of Table 1), the Δ*E*_HB_ values for the halogen substituents clearly decrease in magnitude down the group (from 4.7 kcal mol^–1^ for F to 3.5 kcal mol^–1^ for Br). Similarly, for the pnictogens, the magnitude of Δ*E*_HB_ is smaller for A = PHCH_3_ and P­(CH_3_)_2_ than for A = NHCH_3_ and N­(CH_3_)_2_ (approximately 2.6 to 4.0 kcal mol^–1^ vs 7.4 to 8.2 kcal mol^–1^). This decreasing trend down groups of the periodic table extends to the simplest chalcogen-containing hydrogen bond acceptors, with the magnitude of Δ*E*_HB_ for SH being less than one-third that for OH (2.0 vs 6.8 kcal mol^–1^, respectively). However, that difference is attenuated for the gauche conformer of SCH_3_, where the magnitude of Δ*E*_HB_ increases to 6.1 kcal mol^–1^, which is comparable to the values for both OCH_3_ conformers (6.8 kcal mol^–1^ for anti and 5.1 kcal mol^–1^ for gauche). This observation is consistent with previous findings for both intra- and intermolecular systems,^89–91^ where OH···S interactions can be as strong as their OH···O counterparts depending on the molecular framework and local electronic environment as noted by Grabowski and co-workers.^89^ This further demonstrates that sulfur should no longer be overlooked as a potential hydrogen bond acceptor, as its participation may play a significant role in biological processes such as protein folding.^91,92^

The M06-2X, df-MP2, and PNO-LCCSD­(T)-F12 ΔE_HB_ values are quite consistent. Comparing the results obtained with the TZ basis set, both the M06-2X and df-MP2 relative energies are typically within about 0.2 kcal mol^–1^ of the PNO-LCCSD­(T)-F12 values, but the deviations are usually in opposite directions (i.e., over- vs underestimation). As seen in the second and third columns of data, the addition of diffuse functions has a small effect. The magnitude of df-MP2 ΔE_HB_ values increases by ca. 0.1 kcal mol^–1^ when the aTZ basis set is used instead of TZ. The PNO-LCCSD­(T)-F12/TZ and PNO-LCCSD­(T)-F12/TZ-F12 single-point energies (last two columns of Table 1) are generally within about a 10th of a kcal mol^–1^, with differences exceeding 0.2–0.3 kcal mol^–1^ for some of the larger O- and N-containing hydrogen bond acceptor groups.

### 3.2 Geometric Analysis

Table 2 reports a few relevant geometrical parameters computed at the df-MP2/TZ and M06-2X/TZ levels of theory for the +HB and −HB norbornane systems identified in this work. The trend in values is consistent across both levels of theory. The covalent O–H bond lengths for the −HB conformations serve as reference values for representing the absence of an OH···A interaction. In these noninteracting conformations, the O–H bond lengths fall within a narrow range of 0.960 to 0.964 Å at df-MP2/TZ and 0.959 to 0.962 Å at M06-2X/TZ levels of theory. Rotation of the OH group to form the intramolecular hydrogen bond in the +HB conformation leads to an elongation of the O–H bond length (Δ*R*) for every acceptor group. The maximum Δ*R* reaches +0.015 Å at df-MP2/TZ and +0.011 Å at M06-2X/TZ levels of theory, this is observed for OH···N interactions. These bond length changes induced by the intramolecular OH···A contacts are consistent with previous studies within this group^28,38^ and with the 2011 IUPAC hydrogen bond definition.^4^ The associated changes in O–H stretching frequencies along with their trends across different functional groups are discussed in the following section.

**Table 2:** Selected Geometric Parameters Computed at the df-MP2/TZ and M06-2X/TZ Levels of Theory: Covalent O–H Bond Lengths for the ±HB Conformations (*R*_±HB_), the Corresponding Bond Elongation Induced by the Formation of OH···A Intramolecular Contacts (Δ*R*) as Well as the OH···A Distances (*R*) and Angles (θ) for the +HB Conformations^a^

	df-MP2/TZ					M06-2X/TZ				
Acceptor	*R*_–HB_	*R*_+HB_	Δ*R*	R(OH···A)	θ(OH···A)	*R*_–HB_	*R*_+HB_	Δ*R*	R(OH···A)	θ(OH···A)
F	0.962	0.963	+0.001	1.935	138	0.960	0.961	+0.001	1.957	135
Cl	0.962	0.964	+0.002	2.270	142	0.960	0.961	+0.002	2.323	138
Br	0.962	0.965	+0.002	2.373	143	0.960	0.962	+0.002	2.449	137
OH	0.962	0.967	+0.004	1.868	142	0.960	0.964	+0.004	1.905	140
OCH_3_-anti	0.962	0.967	+0.005	1.859	143	0.960	0.964	+0.004	1.907	140
OCH_3_-gauche	0.962	0.968	+0.007	1.909	141	0.960	0.964	+0.005	1.996	134
SH	0.962	0.966	+0.004	2.336	143	0.960	0.963	+0.003	2.408	122
SCH_3_-anti	0.964	0.967	+0.003	2.312	129	0.962	0.963	+0.002	2.384	124
SCH_3_-gauche	0.961	0.970	+0.009	2.270	141	0.959	0.966	+0.007	2.350	135
NHCH_3_-anti	0.961	0.975	+0.014	1.892	142	0.959	0.969	+0.010	1.966	137
NHCH_3_-gauche	0.962	0.977	+0.015	1.861	144	0.960	0.971	+0.011	1.933	140
N(CH_3_)_2_	0.962	0.977	+0.015	1.883	144	0.960	0.970	+0.011	1.974	139
PH_2_	0.962	0.966	+0.004	2.386	126	0.960	0.963	+0.003	2.469	119
PHCH_3_-anti	0.963	0.968	+0.005	2.350	129	0.961	0.964	+0.003	2.429	123
PHCH_3_-gauche	0.962	0.968	+0.006	2.352	125	0.960	0.964	+0.004	2.440	119
P(CH_3_)_2_	0.962	0.969	+0.007	2.330	128	0.960	0.965	+0.005	2.416	122

a All bond lengths are reported in Å and angles in degrees.

In addition, Table 2 also reports the df-MP2/TZ and M06-2X/TZ optimized length of OH···A contacts (R­(OH···A)) for all +HB conformations characterized in this work. At both levels of theory, a trend is observed down the halogen group in which the R­(OH···A) increases from approximately 1.9 Å (F) to 2.4 Å (Br), which is consistent with the changes in their atomic radii. A similar increase is observed for the pnictogen- and chalcogen-containing hydrogen bond acceptor groups, R­(OH···A) ≈1.9 Å for A = N or O, and 2.2–2.4 Å for A = P or S. The angles (θ­(OH···A)) about the intramolecular OH···A contacts are listed in Table 2, which shows they are appreciably bent, hovering around 135° (±15°). Crystallographic precedents for intramolecular hydrogen bonding in rigid molecular frameworks can be found in several bicyclic and polycyclic systems deposited in the Cambridge Structural Database.^93^ Structures of rigid, conformationally constrained molecules^94–96^ feature intramolecular OH···A contact distances on the order of 1.7 to 1.9 Å for A = O and N. These solid-state metrics are consistent with the corresponding df-MP2 and M06-2X optimized OH···O and OH···N distances reported in Table 2. The Cartesian coordinates of all of the optimized +HB and −HB minima are available in the Supporting Information.

### 3.3 Spectroscopic and QTAIM Analysis

The first column of numerical data in Table 3 reports the M06-2X/TZ harmonic O–H stretching frequencies for the −HB conformations identified in this work. These ω_–HB_ values serve as a reference for systems without the OH···A interaction and fall within a fairly narrow range (e.g., 3883 ± 18 cm^–1^). Potential inductive effects and/or repulsive interactions were examined by replacing the hydrogen bond acceptor group with a single H atom and recomputing the optimized structure and harmonic vibrational frequencies for the −HB conformation. The O–H stretching frequency for this additional reference structure lacking an intramolecular OH···A contact is 3894 cm^–1^, and that result is remarkably consistent with the ω_–HB_ values in the first column of data in Table 3 (within 8 cm^–1^ for every system except the anti-conformations of A = SCH_3_ and PHCH_3_). This relatively close agreement suggests that the apparent stabilizing effect of the formation of the intramolecular OH···A contact relative to the −HB conformation is not significantly exaggerated by potential repulsive interactions or inductive effects within the −HB conformation.

**Table 3:** M06-2X/TZ Harmonic O–H Stretching Frequencies and Isotropic NMR Chemical Shielding Constants for the Hydroxyl H Atom for the −HB Conformations (ω_–HB_ in cm^–1^ and σ_–HB_ in ppm, Respectively) and Corresponding Shifts Induced by the Formation of OH···A Intramolecular Contacts (Δω and Δσ) along with Electron Density Values (ρ­(*r*) in *e* bohr^–3^) Determined at the BCP Along the OH···A Contacts in the +HB Conformations at the Same Level of Theory

acceptor	ω_–HB_	Δω	σ_–HB_	Δσ	ρ(*r*)_+HB_
F	3888	–6	31.36	–1.79	0.0217
Cl	3886	–27	31.33	–2.06	0.0197
Br	3886	–39	31.32	–2.03	0.0187
OH	3886	–63	31.47	–3.40	0.0273
OCH_3_-anti	3886	–67	31.51	–3.10	0.0270
OCH_3_-gauche	3889	–74	31.51	–2.77	0.0246
SH	3890	–61	31.34	–1.29	0.0184
SCH_3_-anti	3866	–49	31.38	–1.37	0.0190
SCH_3_-gauche	3897	–133	31.09	–3.12	0.0223
NHCH_3_-anti	3901	–211	31.23	–3.84	0.0300
NHCH_3_-gauche	3888	–237	31.66	–5.37	0.0330
N(CH_3_)_2_	3888	–221	31.49	–4.45	0.0298
PH_2_	3892	–62	31.43	–1.02	0.0178
PHCH_3_-anti	3877	–76	31.63	–1.68	0.0189
PHCH_3_-gauche	3890	–84	31.25	–1.22	0.0188
P(CH_3_)_2_	3889	–106	31.34	–1.69	0.0195

The second column of data in Table 3 shows the O–H stretching frequency shifts (Δω) induced by the rotation of the OH donor toward the acceptor group, resulting in the formation of an intramolecular hydrogen bond. It has been demonstrated in several other studies involving intramolecular systems^28,38,97–101^ that when OH groups are oriented toward an hydrogen bond acceptor group, the harmonic vibrational stretching frequency associated with the O–H bond shifts to a lower energy relative to the corresponding non-hydrogen-bonded orientation, commonly referred to as a “red shift”, which is a distinguishing characteristic of hydrogen bond formation.^4^ As you move down the pnictogen-containing acceptors, Δω decreases in magnitude by a factor of 2 or more from the N-containing groups (with shifts exceeding −200 cm^–1^) to the P-analogues (with shifts ranging from −62 cm^–1^ to −106 cm^–1^). The chalcogen-containing hydrogen bond acceptors exhibit mixed results: Δω decreases in magnitude for the anti-conformations of OCH_3_ and SCH_3_ (−67 and −49 cm^–1^, respectively) but increases for the gauche conformations of OCH_3_ and SCH_3_ (−74 and −133 cm^–1^, respectively). The OH and SH acceptors showed very minor differences, staying within −2 cm^–1^. In contrast, as you move down the group of halogen hydrogen bond acceptors, Δω increases in magnitude from −6 cm^–1^ (F) to −39 cm^–1^ (Br). Overall, these observations are consistent with the recent work of Wategaonkar and co-workers, which demonstrated that the shifts in the O–H stretching frequencies are not necessarily correlated with the hydrogen bond strengths.^92,102^ This relationship is plotted in the top panel of Figure 3, and the correlation is rather weak (*R* ^2^ = 0.41).

**Figure 3:**
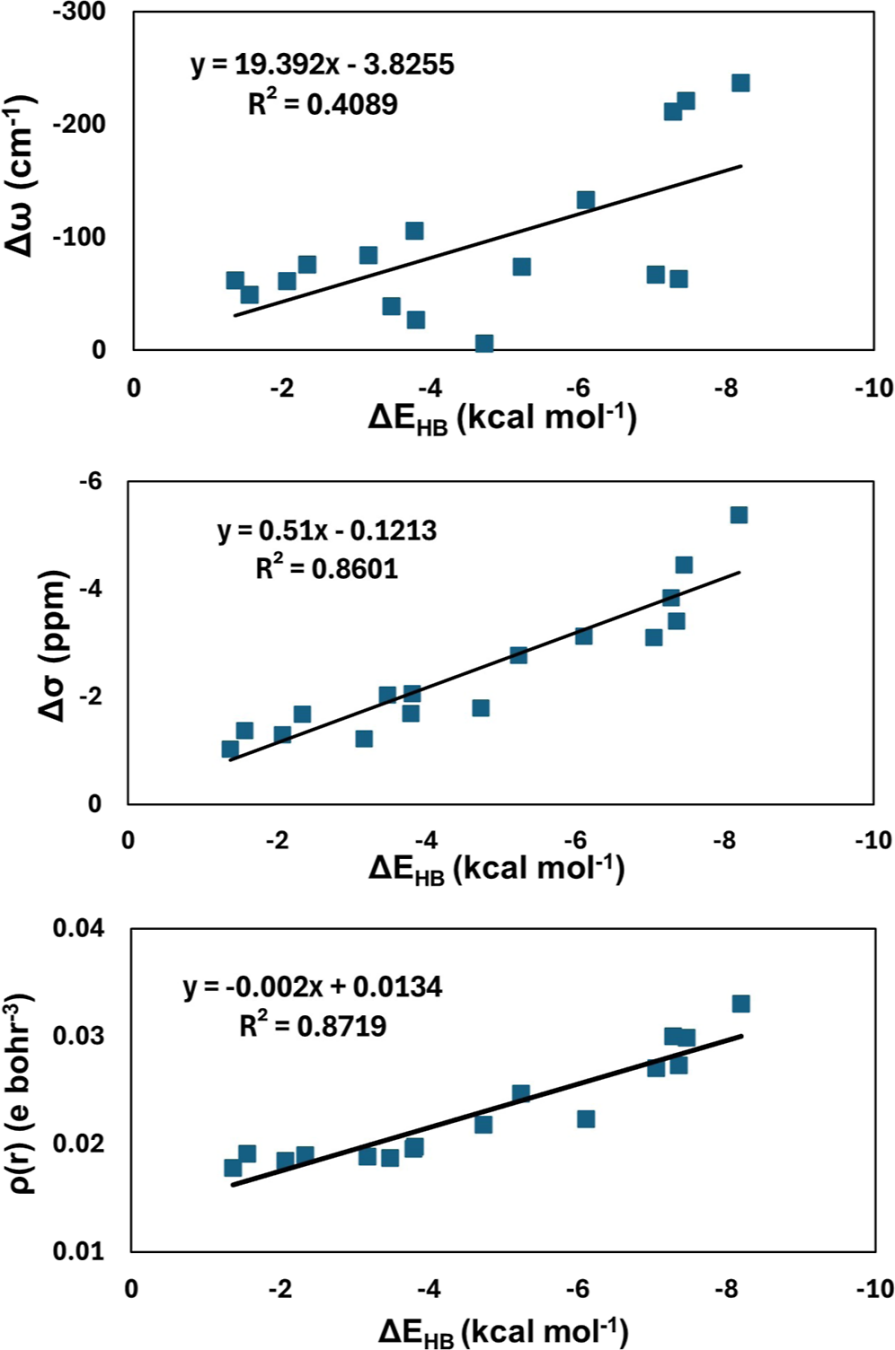
Relationship between the hydrogen bond energy (Δ*E*_HB_) of 2,6-disubstituted +HB conformations and (top) shifts in O–H stretching frequency (Δω), (middle) shifts in isotropic NMR chemical shielding constant (Δσ), and (bottom) electron density (ρ­(*r*)) at the BCP associated with the OH···A interaction, calculated at the M06-2X/TZ level of theory.

Columns 3 and 4 of numerical data in Table 3 show the M06-2X/TZ isotropic NMR chemical shielding constants (σ_–HB_) for the H atom in the OH donor group for all −HB conformations identified in this work, followed by the shifts (Δσ) induced by the rotation of the OH donor into the +HB conformation (σ_+HB_), resulting in the formation of the intramolecular hydrogen bond. In every case, the σ_+HB_ values are smaller than the σ_–HB_ values, suggesting that the hydroxyl H atom is consistently deshielded in the intramolecular hydrogen-bonded conformation. The largest shift (Δσ) induced by the formation of the intramolecular OH···A contact is obtained for NHCH_3_ and has a magnitude well above 5 ppm, whereas the smallest shift observed is for PH_2_ with a magnitude that barely exceeds 1 ppm. Interestingly, the same trend is reflected in the ΔE_HB_ values calculated at the M06-2X/TZ level of theory (largest magnitude for NHCH_3_ and smallest magnitude for PH_2_). The relationship between Δσ and ΔE_HB_ illustrated in the middle panel of Figure 3 exhibits a modest correlation between these two parameters (R^2^ = 0.86). This suggests that Δσ may be a useful indicator of the ability of these types of hydrogen bond acceptor groups to form attractive intramolecular interactions with a proximal OH group on similar molecular scaffolds. Many of the general spectroscopic trends discussed in this section were also observed for OH hydrogen bonds to O, N, S and P bridges in (semi)­rigid cyclohexanol systems despite significantly longer hydrogen bond distances (by ≈0.4 Å).^38^ For example, that study and this work both show that the spectroscopic shifts for the OH donor (Δω and Δσ) are often quite similar when the hydrogen bond acceptor is an O or S on these carbon skeletons, whereas N and P typically exhibit far more pronounced differences.

Quantum Theory of Atoms in Molecules (QTAIM) analysis is a widely used topological approach based on electron density (ρ­(*r*)) that enables quantitative characterization of intramolecular interactions.^54,72,73^ To evaluate how the various acceptors influence the strength of the intramolecular OH···A interactions in these substituted norbornane systems, M06-2X/TZ electron density values were determined at the BCPs for all of the unique +HB conformations. The BCPs are depicted as orange spheres in Figure 2 (lying along the intramolecular hydrogen bonds) and are reported in the last column of Table 3 (ρ­(*r*) in *e* bohr^–3^). For all hydrogen bond acceptor groups, the +HB conformations exhibit electron densities within the typical hydrogen bonding range (0.002 to 0.040 *e* bohr^–3^),^103,104^ ranging from ρ­(*r*) = 0.0178 *e* bohr^–3^ for PH_2_ to 0.0330 *e* bohr^–3^ for the gauche NHCH_3_ conformation.^54,72^ These values reported in Table 3 are consistent with the relative energetic results discussed above (Table 1). For example, the N-containing hydrogen bond acceptor groups exhibit the largest ρ­(*r*) values (from 0.0300 to 0.0330 *e* bohr^–3^) and the most negative ΔE_HB_ results near the CCSD­(T) CBS limit (from −7.4 to −8.2 kcal mol^–1^). Similarly, the trend in ΔE_HB_ for the three halogen acceptors (F, Cl, and Br) is reflected in the corresponding electron density data (largest for *F* with ρ­(*r*) = 0.0217 *e* bohr^–3^ and smallest for Br with ρ­(*r*) = 0.0187 *e* bohr^–3^). Further, the relationship between ρ­(*r*) and Δ*E*_HB_, as shown in the bottom panel of Figure 3 also demonstrates a modest correlation with an *R* ^2^ value of 0.87. Therefore, ρ­(*r*) may also provide a useful metric for comparing the capacity of these functional groups to accept an intramolecular OH···A hydrogen bond in similar molecular and electronic environments. Additionally, the Laplacian of the electron density (∇^2^ρ­(*r*)), the total energy density (*H*(*r*)), and the −*G*(*r*)/*V*(*r*) ratio derived from QTAIM analysis, serve as indicators of the nature of OH···A hydrogen bonding interactions.^105,106^ The corresponding values, computed at the M06-2X/TZ level of theory for the +HB conformations, are listed in Table S1. The results suggest that most of the OH···A hydrogen bonds are closed-shell interactions.

## 4 Conclusions

A total of 32 2,6-disubstituted norbornane minima were characterized to investigate the ability of various hydrogen bond acceptor groups (F, Cl, Br, OH, OCH_3_, SH, SCH_3_, NHCH_3_, N­(CH_3_)_2_, PH_2_, PHCH_3_, and P­(CH_3_)_2_) to form an intramolecular hydrogen bond with a nearby OH donor. Some of the key outcomes are summarized below.

- M06-2X, df-MP2, and PNO-LCCSD­(T)-F12 results consistently reveal attractive OH···A interactions when the OH donor is oriented toward the adjacent acceptor group, lowering the electronic energy relative to the non-hydrogen-bonded (−HB) conformations by ≈2 kcal mol^–1^ to more than 8 kcal mol^–1^ across all levels of theory.
- In every case, the formation of the intramolecular hydrogen bond elongated the O–H covalent bonds (up to 0.015 Å) for the +HB structures relative to their −HB counterparts and shifted the corresponding OH stretching frequencies to lower energies, typically in the range of ca. −50 to −100 cm^–1^ (for hydrogen bond acceptor groups with more than a single atom), and in some cases exceeding −200 cm^–1^.
- Isotropic NMR chemical shielding constants also showed significant deshielding of the donor H atom in all +HB structures, with shifts ranging from −1.02 to −5.37 ppm, indicating significant polarization of the O–H bond by the OH···A interaction.
- QTAIM analysis further confirmed the presence of bond critical points (BCPs) in all +HB conformers, with electron density (ρ­(*r*)) values at the BCPs ranging from 0.0178 to 0.0330 *e* bohr^–3^, consistent with the typical range for hydrogen bonds.
- Additionally, there is a fair correlation of Δ*E*_HB_ with both Δσ and ρ­(*r*), with the *R* ^2^ exceeding 0.85 in each case. The changes in structural, energetic, and spectroscopic parameters for the different substituents underscore the robustness of OH···A interactions on the norbornane framework.
- Although all metrics indicate the proximal OH···N interactions have the most pronounced impact, OH···A contacts involving P and S hydrogen bond acceptor groups can also be significant in terms of the energetics and other parameters examined in this study.

## Supplementary Material



## Notes

Cartesian coordinates of all minima optimized at the df-MP2/TZ and M06-2X/TZ levels of theory (PDF)
