# Supplementary material for: Probing Proximal Intramolecular Hydrogen Bonding Interactions on a Norbornane Scaffold
Source: J Phys Chem A. 2026 Jan 28;130(5):1125–34. doi: 10.1021/acs.jpca.5c08010 (PMC12884526; doi:10.1021/acs.jpca.5c08010)
Supplement: Supplementary file 1 [file jp5c08010_si_001.pdf]

Supporting Information for:

Probing Proximal Intramolecular Hydrogen Bonding Interactions on a Norbornane Scaffold

Carly A. Rock,[†] Dakota B. Green,[‡] Martin J. Flores,[‡] Jeremy M. Carr,[‡] Thufail M. Ismail[¶] and Gregory S. Tschumper[*,¶]

†Oxford High School, Oxford, MS, 38655, USA

‡Central Alabama Community College, Alexander City, AL, 35010, USA

¶Department of Chemistry, Missouri University of Science and Technology, Rolla, MO, 65409, USA

I. ADDITIONAL QTAIM RESULTS

TABLE S1: M06-2X/TZ QTAIM parameters (in a.u.) at the bond critical points along the OH$\cdots$A interaction in the +HB conformations, the Laplacian of electron density ($\nabla^2\rho(r)$), the total energy density H(r), and the $-G(r)/V(r)$ ratio.

**Acceptor**	$\nabla^2\rho(r)$	$H(r)$	$-G(r)/V(r)$
F	0.1021	+0.0035	1.1856
Cl	0.0632	+0.0009	1.0680
Br	0.0533	+0.0005	1.0393
OH	0.1109	+0.0017	1.0686
OCH_3-anti	0.1090	+0.0016	1.0686
OCH_3-gauche	0.0979	+0.0022	1.1110
SH	0.0564	+0.0010	1.0874
SCH_3-anti	0.0569	+0.0009	1.0699
SCH_3-gauche	0.0560	−0.0003	0.9763
$NHCH_3$-anti	0.0899	−0.0010	0.9605
$NHCH_3$-gauche	0.0900	−0.0026	0.9067
$N(CH_3)_2$	0.0858	−0.0013	0.9462
PH_2	0.0468	+0.0005	1.0489
$PHCH_3$-anti	0.0474	+0.0002	1.0202
$PHCH_3$-gauche	0.0487	+0.0004	1.0309
$P(CH_3)_2$	0.0485	+0.0001	1.0122

II. +HB CONFORMATIONS

TABLE S2: Cartesian coordinates in Angstroms (Å) for the M06-2X/TZ optimized 2,6-disubstituted norbornane +HB conformation with an OH donor and F acceptor group.

Atom	x	y	z
O	2.123472	0.758428	-0.101800
H	2.150711	-0.137851	-0.447505
F	1.068124	-1.750496	-0.206062
C	-1.385450	0.594813	0.992139
C	-1.436835	0.504897	-0.547227
C	-1.165508	-0.990595	-0.743351
C	-0.129148	-1.293678	0.357164
C	0.042345	0.032751	1.102515
C	0.794873	1.062548	0.237303
C	-0.174354	1.280559	-0.959399
H	0.268080	0.891394	-1.876914
H	-0.378407	2.339169	-1.112866
H	0.859015	1.982786	0.818989
H	-1.460488	1.612043	1.375328
H	-2.138390	-0.025610	1.479142
H	0.458190	-0.075454	2.100955
H	-0.455305	-2.085753	1.030029
H	-2.069344	-1.580324	-0.593835
H	-0.756678	-1.234575	-1.723006
H	-2.353899	0.852887	-1.015098

TABLE S3: Cartesian coordinates in Angstroms (Å) for the df-MP2/TZ optimized 2,6-disubstituted norbornane +HB conformation with an OH donor and F acceptor group.

Atom	x	y	z
O	2.069754	0.803554	-0.121949
H	2.077286	-0.106501	-0.436260
F	1.025098	-1.715698	-0.213895
C	-1.430081	0.622555	1.007355
C	-1.500205	0.529763	-0.529768
C	-1.223632	-0.962792	-0.724340
C	-0.171766	-1.258026	0.362174
C	0.000762	0.068169	1.102344
C	0.735449	1.099687	0.228921
C	-0.246007	1.306046	-0.958387
H	0.188459	0.910337	-1.876341
H	-0.454112	2.363115	-1.116769
H	0.797806	2.022124	0.806493
H	-1.503849	1.640182	1.390478
H	-2.174736	-0.000482	1.504109
H	0.427282	-0.034847	2.096733
H	-0.488070	-2.048108	1.041025
H	-2.122041	-1.556597	-0.559510
H	-0.827597	-1.206610	-1.708934
H	-2.424532	0.872968	-0.987569

TABLE S4: Cartesian coordinates in Angstroms (Å) for the df-MP2/aTZ optimized 2,6-disubstituted norbornane +HB conformation with an OH donor and F acceptor group.

Atom	x	y	z
O	2.070878	0.805583	-0.121750
H	2.088709	-0.105916	-0.436420
F	1.027591	-1.718047	-0.212053
C	-1.432527	0.623378	1.005747
C	-1.500128	0.529477	-0.532172
C	-1.222319	-0.963817	-0.725919
C	-0.174424	-1.258538	0.364987
C	-0.000369	0.068442	1.103720
C	0.733554	1.100345	0.229473
C	-0.245692	1.307028	-0.960050
H	0.189988	0.911534	-1.878464
H	-0.454316	2.365119	-1.116680
H	0.797979	2.023049	0.807964
H	-1.505962	1.642074	1.388529
H	-2.177313	-0.001047	1.502425
H	0.425571	-0.033206	2.099588
H	-0.487906	-2.050952	1.043642
H	-2.121845	-1.558001	-0.562907
H	-0.822529	-1.207583	-1.709984
H	-2.424832	0.871698	-0.992219

TABLE S5: Cartesian coordinates in Angstroms (Å) for the M06-2X/TZ optimized 2,6-disubstituted norbornane +HB conformation with an OH donor and Cl acceptor group.

Atom	x	y	z
O	0.319705	2.227341	-0.074156
H	1.065206	1.759430	-0.461095
Cl	2.217818	-0.224120	-0.098109
C	-1.641433	-0.710316	0.958204
C	-1.553148	-0.816100	-0.578143
C	-0.189149	-1.497921	-0.743968
C	0.624867	-0.899185	0.429099
C	-0.332777	0.090996	1.102612
C	-0.695566	1.307805	0.226504
C	-1.402873	0.656712	-0.994490
H	-0.791246	0.770460	-1.890355
H	-2.365025	1.128131	-1.188984
H	-1.426280	1.885504	0.793855
H	-2.509192	-0.151770	1.307781
H	-1.616217	-1.679911	1.455851
H	-0.025673	0.363705	2.109027
H	0.906191	-1.665987	1.144342
H	-0.262897	-2.579378	-0.633110
H	0.272009	-1.287669	-1.706564
H	-2.367890	-1.342973	-1.067649

TABLE S6: Cartesian coordinates in Angstroms (Å) for the df-MP2/TZ optimized 2,6-disubstituted norbornane +HB conformation with an OH donor and Cl acceptor group.

Atom	x	y	z
O	0.252371	2.210779	-0.072476
H	1.006940	1.709385	-0.401698
Cl	2.132133	-0.238593	-0.100627
C	-1.716562	-0.727258	0.956890
C	-1.629087	-0.834894	-0.578093
C	-0.266413	-1.514890	-0.743803
C	0.547433	-0.915287	0.428801
C	-0.409095	0.073160	1.101897
C	-0.769272	1.287089	0.226463
C	-1.472894	0.635127	-0.995294
H	-0.855646	0.743967	-1.887212
H	-2.432471	1.110012	-1.194418
H	-1.501967	1.865523	0.790140
H	-2.583898	-0.167032	1.306222
H	-1.691331	-1.696640	1.455806
H	-0.104209	0.345076	2.109603
H	0.824862	-1.683646	1.145608
H	-0.337681	-2.596839	-0.634406
H	0.193805	-1.299430	-1.705816
H	-2.444967	-1.361139	-1.067455

TABLE S7: Cartesian coordinates in Angstroms (Å) for the df-MP2/aTZ optimized 2,6-disubstituted norbornane +HB conformation with an OH donor and Cl acceptor group.

Atom	x	y	z
O	0.246010	2.210023	-0.087839
H	1.011096	1.712060	-0.402593
Cl	2.132720	-0.230183	-0.101049
C	-1.713259	-0.730129	0.963058
C	-1.626710	-0.844544	-0.572341
C	-0.260023	-1.518394	-0.736681
C	0.551140	-0.911319	0.433976
C	-0.408061	0.077136	1.103095
C	-0.775007	1.284352	0.220981
C	-1.479344	0.624931	-0.996902
H	-0.864953	0.733916	-1.891733
H	-2.443017	1.094664	-1.193062
H	-1.506129	1.867253	0.783698
H	-2.583035	-0.171161	1.311030
H	-1.681220	-1.697788	1.466788
H	-0.103186	0.356212	2.109881
H	0.835151	-1.674915	1.154643
H	-0.326114	-2.601140	-0.624018
H	0.201145	-1.301450	-1.698900
H	-2.440784	-1.377981	-1.059098

TABLE S8: Cartesian coordinates in Angstroms (Å) for the M06-2X/TZ optimized 2,6-disubstituted norbornane +HB conformation with an OH donor and Br acceptor group.

Atom	x	y	z
O	-0.299544	2.266373	-0.058989
H	0.472313	1.859696	-0.463784
Br	1.918660	-0.071556	-0.045344
C	-2.094317	-0.787207	0.937525
C	-1.966931	-0.892820	-0.595825
C	-0.562470	-1.495991	-0.732168
C	0.184991	-0.855888	0.463010
C	-0.835282	0.088610	1.106487
C	-1.255284	1.279693	0.219378
C	-1.892162	0.584886	-1.015548
H	-1.265677	0.730175	-1.896655
H	-2.874484	0.999339	-1.236926
H	-2.034275	1.807173	0.771167
H	-2.999246	-0.276367	1.265448
H	-2.025452	-1.751582	1.440843
H	-0.566865	0.381541	2.118176
H	0.484452	-1.604562	1.188576
H	-0.575224	-2.580211	-0.622990
H	-0.098182	-1.259522	-1.686789
H	-2.739282	-1.467167	-1.100437

TABLE S9: Cartesian coordinates in Angstroms (Å) for the df-MP2/TZ optimized 2,6-disubstituted norbornane +HB conformation with an OH donor and Br acceptor group.

Atom	x	y	z
O	-0.444382	2.252163	-0.056785
H	0.345767	1.806760	-0.385020
Br	1.739636	-0.082508	-0.043144
C	-2.251118	-0.800876	0.934478
C	-2.123155	-0.907353	-0.597546
C	-0.719511	-1.507127	-0.734316
C	0.026461	-0.867697	0.462140
C	-0.993956	0.073726	1.105666
C	-1.408680	1.262913	0.219535
C	-2.042445	0.568365	-1.016557
H	-1.410667	0.710206	-1.893646
H	-3.022519	0.986593	-1.241294
H	-2.189295	1.792553	0.766668
H	-3.156531	-0.289115	1.261213
H	-2.182180	-1.765399	1.438500
H	-0.728044	0.364944	2.119051
H	0.317120	-1.620998	1.189180
H	-0.728595	-2.592129	-0.630400
H	-0.256777	-1.261467	-1.687714
H	-2.896297	-1.480941	-1.102933

TABLE S10: Cartesian coordinates in Angstroms (Å) for the df-MP2/aTZ optimized 2,6-disubstituted norbornane +HB conformation with an OH donor and Br acceptor group.

Atom	x	y	z
O	-0.438424	2.248085	-0.054081
H	0.365368	1.802221	-0.352850
Br	1.733199	-0.076918	-0.052334
C	-2.247241	-0.804559	0.942484
C	-2.119165	-0.912642	-0.590375
C	-0.715402	-1.513307	-0.724738
C	0.030134	-0.868678	0.468256
C	-0.989906	0.071424	1.112741
C	-1.406113	1.258727	0.225051
C	-2.037192	0.563125	-1.012300
H	-1.401383	0.703829	-1.887607
H	-3.017366	0.982033	-1.239213
H	-2.186394	1.791767	0.771043
H	-3.153372	-0.292598	1.269664
H	-2.174880	-1.769295	1.447413
H	-0.722506	0.364285	2.126281
H	0.330546	-1.618024	1.196686
H	-0.724878	-2.598620	-0.616186
H	-0.248443	-1.268730	-1.677217
H	-2.892861	-1.487144	-1.095887

TABLE S11: Cartesian coordinates in Angstroms (Å) for the M06-2X/TZ optimized 2,6-disubstituted norbornane +HB conformation with an OH donor and OH acceptor group.

Atom	x	y	z
C	-1.511129	-0.265533	-0.545587
C	-0.407147	-1.253146	-0.957759
C	0.595753	-1.198819	0.231430
C	0.031703	-0.055226	1.096794
C	0.094949	1.291728	0.361991
C	-0.980616	1.158051	-0.749847
H	-0.538486	1.303456	-1.736275
H	-1.771652	1.896466	-0.616936
H	-0.173306	2.088730	1.060725
O	1.406823	1.542864	-0.134877
H	1.407686	2.378605	-0.606394
C	-1.470268	-0.362215	0.993255
H	-2.101298	0.379943	1.483543
H	-1.721874	-1.351841	1.373925
H	0.463691	-0.021210	2.094104
H	0.507814	-2.117396	0.813810
O	1.951349	-1.114429	-0.124034
H	2.124907	-0.203495	-0.386134
H	-0.797029	-2.260411	-1.099078
H	0.092117	-0.956402	-1.880465
H	-2.476818	-0.448529	-1.010342

TABLE S12: Cartesian coordinates in Angstroms (Å) for the df-MP2/TZ optimized 2,6-disubstituted norbornane +HB conformation with an OH donor and OH acceptor group.

Atom	x	y	z
O	1.377560	1.542139	-0.129606
H	1.356204	2.361618	-0.633063
O	1.915712	-1.101068	-0.128332
H	2.060797	-0.178397	-0.376845
C	-1.550960	-0.260532	-0.552238
C	-0.448489	-1.248072	-0.959656
C	0.554188	-1.190692	0.228527
C	-0.010696	-0.049207	1.092071
C	0.054012	1.294312	0.357745
C	-1.014552	1.158725	-0.758668
C	-1.511733	-0.354677	0.985449
H	-0.566066	1.295423	-1.743334
H	-1.802522	1.901710	-0.633262
H	-0.218776	2.092180	1.053107
H	-2.143073	0.388566	1.474143
H	-1.763793	-1.344127	1.367047
H	0.417477	-0.015766	2.091031
H	0.469426	-2.109190	0.810736
H	-0.837958	-2.256065	-1.097132
H	0.051027	-0.953178	-1.882265
H	-2.516607	-0.441750	-1.018616

TABLE S13: Cartesian coordinates in Angstroms (Å) for the df-MP2/aTZ optimized 2,6-disubstituted norbornane +HB conformation with an OH donor and OH acceptor group.

Atom	x	y	z
O	1.384902	1.538243	-0.126916
H	1.380830	2.377447	-0.599994
O	1.911661	-1.112279	-0.138013
H	2.072702	-0.189436	-0.381612
C	-1.553779	-0.251882	-0.550365
C	-0.456734	-1.243991	-0.964008
C	0.547679	-1.194176	0.223331
C	-0.008203	-0.052269	1.092566
C	0.059183	1.293839	0.362987
C	-1.011167	1.166296	-0.753361
C	-1.512042	-0.351378	0.987685
H	-0.562938	1.304812	-1.738659
H	-1.796747	1.912248	-0.623292
H	-0.206880	2.091589	1.062034
H	-2.138287	0.394693	1.480506
H	-1.766902	-1.341889	1.367048
H	0.422691	-0.025145	2.091596
H	0.462708	-2.114599	0.803917
H	-0.851702	-2.250715	-1.101620
H	0.041733	-0.948712	-1.887985
H	-2.521927	-0.426933	-1.016168

TABLE S14: Cartesian coordinates in Angstroms (Å) for the M06-2X/TZ optimized 2,6-disubstituted norbornane +HB anti conformation with an OH donor and OCH_3 acceptor group.

Atom	x	y	z
C	1.305866	-1.137941	-0.608909
C	1.541930	0.310338	-1.067043
C	1.097206	1.159747	0.159143
C	0.511160	0.098972	1.110596
C	-0.745991	-0.554017	0.518867
C	-0.201855	-1.414767	-0.656059
H	-0.650160	-1.108033	-1.601627
H	-0.416578	-2.472603	-0.504298
H	-1.210370	-1.192892	1.279252
O	-1.700472	0.414443	0.120117
C	-2.953312	-0.143460	-0.194356
H	-3.614564	0.670526	-0.480309
H	-2.880248	-0.853039	-1.023663
H	-3.373821	-0.660736	0.673960
C	1.532393	-1.031581	0.912561
H	1.276406	-1.947042	1.447226
H	2.545270	-0.732389	1.181388
H	0.366168	0.465933	2.123850
H	1.980870	1.577890	0.643860
O	0.278742	2.262369	-0.135362
H	-0.613610	1.929226	-0.282265
H	2.587799	0.490951	-1.312537
H	0.943821	0.573213	-1.939685
H	1.906558	-1.878885	-1.130660

TABLE S15: Cartesian coordinates in Angstroms (Å) for the df-MP2/TZ optimized 2,6-disubstituted norbornane +HB anti conformation with an OH donor and OCH_3 acceptor group.

Atom	x	y	z
O	0.249199	2.227733	-0.151318
H	-0.635975	1.860326	-0.280024
O	-1.700063	0.386222	0.105719
C	-2.949684	-0.194542	-0.214961
H	-3.619450	0.612587	-0.495478
H	-2.865418	-0.896302	-1.047466
H	-3.361274	-0.721376	0.650114
C	1.303296	-1.173958	-0.604607
C	1.522725	0.271299	-1.074134
C	1.078869	1.126594	0.147214
C	0.502013	0.070497	1.105545
C	-0.749396	-0.590052	0.516605
C	-0.200443	-1.461441	-0.646672
C	1.529935	-1.053631	0.914301
H	-0.650553	-1.170053	-1.595911
H	-0.407325	-2.518601	-0.479031
H	-1.220359	-1.220155	1.280204
H	1.281426	-1.967132	1.456191
H	2.540676	-0.743710	1.180256
H	0.355938	0.442410	2.116828
H	1.962185	1.552120	0.625371
H	2.564891	0.460684	-1.328727
H	0.914182	0.521016	-1.943019
H	1.909360	-1.914365	-1.121745

TABLE S16: Cartesian coordinates in Angstroms (Å) for the df-MP2/aTZ optimized 2,6-disubstituted norbornane +HB anti conformation with an OH donor and OCH_3 acceptor group.

Atom	x	y	z
O	0.239175	2.225157	-0.178409
H	-0.649339	1.857651	-0.296704
O	-1.707056	0.380967	0.107122
C	-2.953504	-0.214123	-0.212517
H	-3.628428	0.587378	-0.499436
H	-2.857727	-0.921183	-1.040266
H	-3.358892	-0.738786	0.657581
C	1.309364	-1.173685	-0.593894
C	1.533448	0.268129	-1.072901
C	1.077256	1.131594	0.138788
C	0.501047	0.082965	1.106101
C	-0.747284	-0.587202	0.521744
C	-0.195462	-1.459664	-0.639964
C	1.531904	-1.042022	0.925416
H	-0.642745	-1.166402	-1.591036
H	-0.404014	-2.517369	-0.472767
H	-1.213544	-1.217031	1.289796
H	1.281062	-1.951646	1.474450
H	2.542259	-0.728008	1.191536
H	0.353364	0.463435	2.115041
H	1.953786	1.572357	0.617391
H	2.579740	0.455277	-1.315653
H	0.934603	0.511514	-1.951245
H	1.916634	-1.919100	-1.104494

TABLE S17: Cartesian coordinates in Angstroms (Å) for the M06-2X/TZ optimized 2,6-disubstituted norbornane +HB gauche conformation with an OH donor and OCH_3 acceptor group.

Atom	x	y	z
O	0.575994	2.023784	-0.253800
H	1.053056	1.402681	-0.816316
O	1.665783	-0.484695	-0.598827
C	2.771399	-0.217463	0.238043
H	3.029731	-1.101054	0.829999
H	3.610136	0.037840	-0.405308
H	2.566779	0.620355	0.909709
C	-1.551578	-0.633149	1.136589
C	-1.792573	-0.619315	-0.385489
C	-0.564966	-1.392278	-0.885376
C	0.543906	-0.981069	0.109884
C	-0.163173	0.018583	1.049192
C	-0.510467	1.323832	0.306518
C	-1.593153	0.866654	-0.714110
H	-1.242802	1.026771	-1.734047
H	-2.515668	1.432267	-0.586887
H	-0.951994	2.014472	1.025522
H	-2.266922	-0.028065	1.694066
H	-1.529697	-1.638658	1.558537
H	0.353286	0.183339	1.992665
H	0.873144	-1.843413	0.702095
H	-0.730995	-2.468449	-0.858987
H	-0.275420	-1.119738	-1.899730
H	-2.740920	-1.030337	-0.722354

TABLE S18: Cartesian coordinates in Angstroms (Å) for the df-MP2/TZ optimized 2,6-disubstituted norbornane +HB gauche conformation with an OH donor and OCH_3 acceptor group.

Atom	x	y	z
O	0.555589	1.998369	-0.288538
H	1.052331	1.321877	-0.771683
O	1.665118	-0.477602	-0.591491
C	2.781817	-0.248404	0.256621
H	3.033644	-1.155574	0.811387
H	3.616070	0.023295	-0.383423
H	2.588217	0.564237	0.958512
C	-1.545749	-0.649592	1.163136
C	-1.796141	-0.656067	-0.356092
C	-0.558479	-1.409136	-0.856418
C	0.545786	-0.978606	0.133787
C	-0.168683	0.021826	1.062166
C	-0.530847	1.309134	0.300175
C	-1.622560	0.827191	-0.698885
H	-1.289106	0.984361	-1.724323
H	-2.551025	1.380329	-0.559307
H	-0.966659	2.014396	1.007969
H	-2.266829	-0.048585	1.718194
H	-1.505523	-1.649872	1.596846
H	0.347536	0.205899	2.001910
H	0.886066	-1.831582	0.732907
H	-0.704827	-2.488087	-0.828175
H	-0.276241	-1.131247	-1.871271
H	-2.739316	-1.087477	-0.683148

TABLE S19: Cartesian coordinates in Angstroms (Å) for the df-MP2/aTZ optimized 2,6-disubstituted norbornane +HB gauche conformation with an OH donor and OCH_3 acceptor group.

Atom	x	y	z
O	0.559548	2.001529	-0.293079
H	1.063380	1.327443	-0.775064
O	1.664302	-0.483841	-0.593745
C	2.786311	-0.269111	0.255279
H	3.037814	-1.187950	0.792003
H	3.617323	0.015344	-0.384510
H	2.592187	0.530305	0.973411
C	-1.545674	-0.642009	1.167746
C	-1.800522	-0.649262	-0.351637
C	-0.566299	-1.406691	-0.856286
C	0.541457	-0.980980	0.132461
C	-0.165132	0.024287	1.061968
C	-0.527586	1.312081	0.300425
C	-1.622262	0.833492	-0.697308
H	-1.287914	0.987342	-1.723828
H	-2.548841	1.391174	-0.556880
H	-0.958524	2.020096	1.009769
H	-2.262712	-0.036034	1.724307
H	-1.506967	-1.642843	1.602463
H	0.355095	0.207709	2.000774
H	0.880553	-1.835775	0.731250
H	-0.716435	-2.486003	-0.828584
H	-0.285733	-1.127049	-1.872124
H	-2.747026	-1.077920	-0.675872

TABLE S20: Cartesian coordinates in Angstroms (Å) for the M06-2X/TZ optimized 2,6-disubstituted norbornane +HB conformation with an OH donor and SH acceptor group.

Atom	x	y	z
C	1.565623	-0.833361	-0.569787
C	1.476963	0.650749	-0.957654
C	0.725365	1.291915	0.241865
C	0.338668	0.071481	1.102780
C	-0.645730	-0.884796	0.404391
C	0.180260	-1.462743	-0.778137
H	-0.247983	-1.199225	-1.744425
H	0.220391	-2.550225	-0.712832
H	-0.884944	-1.676585	1.111035
S	-2.248288	-0.123334	-0.045456
H	-2.754430	-1.219399	-0.628494
C	1.632829	-0.750085	0.967610
H	1.579429	-1.723491	1.455045
H	2.510053	-0.214598	1.330951
H	0.029450	0.343207	2.109367
H	1.424735	1.883708	0.833272
O	-0.298372	2.193391	-0.095797
H	-0.909555	1.766653	-0.705001
H	2.461929	1.096748	-1.088845
H	0.920236	0.802355	-1.883753
H	2.372339	-1.376745	-1.055278

TABLE S21: Cartesian coordinates in Angstroms (Å) for the df-MP2/TZ optimized 2,6-disubstituted norbornane +HB conformation with an OH donor and SH acceptor group.

Atom	x	y	z
O	-0.258869	2.170766	-0.105603
H	-0.903154	1.694431	-0.644583
S	-2.198131	-0.155801	-0.048254
H	-2.666632	-1.224050	-0.703826
C	1.612346	-0.860372	-0.576149
C	1.520548	0.621375	-0.963798
C	0.767002	1.262578	0.234089
C	0.382820	0.044308	1.095026
C	-0.596118	-0.915922	0.398731
C	0.227327	-1.484977	-0.788658
C	1.676953	-0.775621	0.960046
H	-0.199660	-1.206489	-1.751440
H	0.261985	-2.573494	-0.734530
H	-0.825227	-1.712393	1.105297
H	1.623266	-1.748517	1.449370
H	2.553401	-0.238060	1.323347
H	0.075788	0.314767	2.103138
H	1.465919	1.855315	0.824611
H	2.504321	1.070622	-1.093898
H	0.962000	0.770568	-1.888344
H	2.419943	-1.404626	-1.060312

TABLE S22: Cartesian coordinates in Angstroms (Å) for the df-MP2/aTZ optimized 2,6-disubstituted norbornane +HB conformation with an OH donor and SH acceptor group.

Atom	x	y	z
O	-0.260367	2.172699	-0.107465
H	-0.919664	1.695772	-0.630640
S	-2.199584	-0.161523	-0.044301
H	-2.654767	-1.219411	-0.727790
C	1.613265	-0.858068	-0.578101
C	1.522223	0.624687	-0.964551
C	0.767694	1.263499	0.234551
C	0.382752	0.044903	1.095189
C	-0.594568	-0.917120	0.398798
C	0.227239	-1.481569	-0.792090
C	1.678172	-0.774957	0.959175
H	-0.202640	-1.197026	-1.752882
H	0.261166	-2.571109	-0.742271
H	-0.821403	-1.715645	1.105329
H	1.623076	-1.749140	1.447733
H	2.554956	-0.236564	1.322968
H	0.075184	0.315341	2.104193
H	1.464193	1.859455	0.826247
H	2.507023	1.074621	-1.091683
H	0.963374	0.776252	-1.889420
H	2.421074	-1.402771	-1.063593

TABLE S23: Cartesian coordinates in Angstroms (Å) for the M06-2X/TZ optimized 2,6-disubstituted norbornane +HB anti conformation with an OH donor and SCH_3 acceptor group.

Atom	x	y	z
O	-0.708837	2.232193	-0.227911
H	0.071153	1.972396	-0.730249
S	1.840039	0.645025	0.160700
C	3.107135	-0.556493	-0.300550
H	2.796223	-1.142873	-1.162800
H	4.005892	0.000087	-0.553582
H	3.321915	-1.217499	0.537843
C	-1.741550	-1.097111	0.943493
C	-1.508604	-1.240687	-0.572789
C	0.017051	-1.409368	-0.633264
C	0.510752	-0.533932	0.554134
C	-0.782996	0.096066	1.101997
C	-1.441208	1.083794	0.116842
C	-1.851442	0.170862	-1.072635
H	-1.292646	0.439018	-1.970504
H	-2.911534	0.274020	-1.300828
H	-2.337095	1.468188	0.605483
H	-2.771866	-0.846674	1.197095
H	-1.435213	-1.974944	1.512431
H	-0.666666	0.507487	2.101789
H	0.919707	-1.174847	1.336752
H	0.310369	-2.449643	-0.490924
H	0.424196	-1.072662	-1.587155
H	-2.057824	-2.037469	-1.068293

TABLE S24: Cartesian coordinates in Angstroms (Å) for the df-MP2/TZ optimized 2,6-disubstituted norbornane +HB anti conformation with an OH donor and SCH_3 acceptor group.

Atom	x	y	z
O	-0.705623	2.187601	-0.261465
H	0.105712	1.891601	-0.695732
S	1.822337	0.601214	0.160804
C	3.081153	-0.600912	-0.319534
H	2.772123	-1.168365	-1.193936
H	3.985175	-0.045847	-0.557064
H	3.288303	-1.278131	0.507345
C	-1.753542	-1.133068	0.937150
C	-1.514883	-1.291954	-0.575339
C	0.009557	-1.458569	-0.630475
C	0.498117	-0.578993	0.555206
C	-0.794792	0.059222	1.090154
C	-1.443313	1.037470	0.092546
C	-1.850633	0.113835	-1.088931
H	-1.284953	0.371412	-1.984821
H	-2.909509	0.219339	-1.322354
H	-2.340611	1.429546	0.571896
H	-2.784931	-0.877953	1.183421
H	-1.450306	-2.005318	1.517020
H	-0.684490	0.478118	2.087996
H	0.899771	-1.217843	1.345211
H	0.305744	-2.497810	-0.485215
H	0.416616	-1.119874	-1.583641
H	-2.062469	-2.093894	-1.065472

TABLE S25: Cartesian coordinates in Angstroms (Å) for the df-MP2/aTZ optimized 2,6-disubstituted norbornane +HB anti conformation with an OH donor and SCH_3 acceptor group.

Atom	x	y	z
O	-0.701127	2.190072	-0.267529
H	0.118446	1.896673	-0.691777
S	1.827230	0.595251	0.161982
C	3.074395	-0.615187	-0.330435
H	2.749834	-1.178545	-1.202659
H	3.979947	-0.064256	-0.574952
H	3.280100	-1.293545	0.496711
C	-1.753682	-1.127964	0.942437
C	-1.518277	-1.288525	-0.571392
C	0.006774	-1.454147	-0.631229
C	0.497745	-0.578261	0.556303
C	-0.792489	0.064233	1.092281
C	-1.442491	1.041181	0.093826
C	-1.856012	0.117124	-1.085740
H	-1.293885	0.374886	-1.984740
H	-2.917131	0.223203	-1.312720
H	-2.336480	1.440463	0.575303
H	-2.785077	-0.870880	1.190373
H	-1.448143	-2.000009	1.523064
H	-0.679484	0.485234	2.089997
H	0.898475	-1.219251	1.346568
H	0.304744	-2.494321	-0.490400
H	0.412341	-1.108971	-1.583883
H	-2.067158	-2.091748	-1.060148

TABLE S26: Cartesian coordinates in Angstroms (Å) for the M06-2X/TZ optimized 2,6-disubstituted norbornane +HB gauche conformation with an OH donor and SCH_3 acceptor group.

Atom	x	y	z
O	0.262979	2.114409	-0.392236
H	0.793767	1.520570	-0.938300
S	1.809989	-0.574195	-0.619535
C	2.782107	0.219151	0.682689
H	2.867265	-0.441779	1.543649
H	3.774250	0.403731	0.278402
H	2.325793	1.163711	0.971736
C	-1.828755	-0.459680	1.199561
C	-2.013863	-0.680397	-0.313492
C	-0.785786	-1.542087	-0.635584
C	0.298254	-0.960801	0.316157
C	-0.434602	0.177257	1.058353
C	-0.797349	1.370078	0.153451
C	-1.772247	0.731447	-0.875360
H	-1.315259	0.706386	-1.865843
H	-2.695315	1.304289	-0.952132
H	-1.343941	2.083291	0.771791
H	-2.560018	0.223362	1.632263
H	-1.825043	-1.389024	1.769385
H	0.056758	0.475240	1.982375
H	0.585357	-1.708955	1.055840
H	-0.963612	-2.593150	-0.411568
H	-0.498329	-1.469300	-1.684924
H	-2.954797	-1.130382	-0.620023

TABLE S27: Cartesian coordinates in Angstroms (Å) for the df-MP2/TZ optimized 2,6-disubstituted norbornane +HB gauche conformation with an OH donor and SCH_3 acceptor group.

Atom	x	y	z
O	0.261392	2.099977	-0.394112
H	0.808454	1.454766	-0.869178
S	1.774930	-0.579778	-0.589387
C	2.769608	0.167095	0.722571
H	2.855447	-0.515741	1.565582
H	3.760295	0.347311	0.312395
H	2.332940	1.110907	1.039150
C	-1.859835	-0.443130	1.217446
C	-2.039156	-0.678222	-0.292722
C	-0.811494	-1.541540	-0.603484
C	0.267384	-0.956065	0.351685
C	-0.464486	0.188864	1.078620
C	-0.813965	1.370304	0.158341
C	-1.788942	0.725103	-0.865985
H	-1.328408	0.687752	-1.853688
H	-2.708961	1.302180	-0.949723
H	-1.357989	2.096972	0.763028
H	-2.591134	0.247215	1.639591
H	-1.860620	-1.366819	1.797263
H	0.020278	0.493995	2.003943
H	0.548667	-1.700999	1.098683
H	-0.988621	-2.592556	-0.377290
H	-0.517426	-1.468425	-1.651200
H	-2.980041	-1.129617	-0.599213

TABLE S28: Cartesian coordinates in Angstroms (Å) for the df-MP2/aTZ optimized 2,6-disubstituted norbornane +HB gauche conformation with an OH donor and SCH_3 acceptor group.

Atom	x	y	z
O	0.265197	2.104836	-0.389095
H	0.824899	1.462805	-0.856951
S	1.779072	-0.576040	-0.579185
C	2.769342	0.132369	0.757838
H	2.840157	-0.575279	1.582541
H	3.764715	0.316043	0.358944
H	2.333880	1.070435	1.095297
C	-1.868068	-0.443765	1.202823
C	-2.039522	-0.671954	-0.310298
C	-0.809692	-1.534063	-0.619271
C	0.263383	-0.955238	0.346495
C	-0.470545	0.187904	1.074311
C	-0.815531	1.373218	0.156751
C	-1.785542	0.734211	-0.876617
H	-1.317909	0.701249	-1.862056
H	-2.705100	1.313388	-0.962006
H	-1.360278	2.100362	0.761751
H	-2.601364	0.246225	1.624317
H	-1.871248	-1.371264	1.778181
H	0.010031	0.489595	2.004096
H	0.540588	-1.704561	1.092263
H	-0.988540	-2.587367	-0.401138
H	-0.507099	-1.452343	-1.664908
H	-2.979627	-1.122249	-0.624177

TABLE S29: Cartesian coordinates in Angstroms (Å) for the M06-2X/TZ optimized 2,6-disubstituted norbornane +HB anti conformation with an OH donor and $NHCH_3$ acceptor group.

Atom	x	y	z
C	1.307791	-1.153284	-0.597655
C	1.648550	0.279310	-1.027959
C	1.143317	1.146413	0.163152
C	0.527744	0.097274	1.113188
C	-0.748911	-0.525242	0.520446
C	-0.215759	-1.331554	-0.696182
H	-0.607413	-0.931773	-1.632418
H	-0.503245	-2.380611	-0.630410
H	-1.153445	-1.229566	1.262466
N	-1.776783	0.446317	0.149130
H	-1.952274	1.048435	0.947064
C	-3.024189	-0.192084	-0.248224
H	-3.782574	0.565518	-0.435457
H	-2.870709	-0.748701	-1.172008
H	-3.402836	-0.892602	0.507606
C	1.511823	-1.066727	0.926998
H	1.211698	-1.971841	1.456003
H	2.533038	-0.808738	1.208250
H	0.394612	0.467577	2.128842
H	1.995230	1.599787	0.671560
O	0.307795	2.218030	-0.196013
H	-0.533394	1.832029	-0.483630
H	2.720632	0.405137	-1.178105
H	1.144790	0.574655	-1.948280
H	1.875117	-1.924352	-1.113430

TABLE S30: Cartesian coordinates in Angstroms (Å) for the df-MP2/TZ optimized 2,6-disubstituted norbornane +HB anti conformation with an OH donor and $NHCH_3$ acceptor group.

Atom	x	y	z
O	0.260659	2.175998	-0.249504
H	-0.584263	1.747303	-0.478368
N	-1.789415	0.422471	0.132307
H	-1.989573	1.011734	0.935461
C	-3.028295	-0.235689	-0.273407
H	-3.794704	0.512361	-0.461749
H	-2.861158	-0.786058	-1.196391
H	-3.399996	-0.941561	0.479195
C	1.293001	-1.193660	-0.567058
C	1.632151	0.228136	-1.024806
C	1.112730	1.120558	0.140901
C	0.504693	0.090983	1.113695
C	-0.766395	-0.546187	0.530862
C	-0.228792	-1.371192	-0.669213
H	-0.613443	-0.983488	-1.613222
H	-0.518690	-2.418565	-0.588767
H	-1.174307	-1.237049	1.283940
C	1.490832	-1.073822	0.954729
H	1.190109	-1.967645	1.502950
H	2.510848	-0.807401	1.233547
H	0.369378	0.481860	2.121425
H	1.957825	1.596625	0.639416
H	2.705462	0.354807	-1.166226
H	1.134618	0.501442	-1.954786
H	1.861981	-1.976261	-1.064220

TABLE S31: Cartesian coordinates in Angstroms (Å) for the df-MP2/aTZ optimized 2,6-disubstituted norbornane +HB anti conformation with an OH donor and $NHCH_3$ acceptor group.

Atom	x	y	z
O	0.257306	2.170478	-0.280228
H	-0.595856	1.742584	-0.487408
N	-1.796403	0.422083	0.141527
H	-2.001593	1.009463	0.945833
C	-3.030220	-0.248787	-0.264049
H	-3.804127	0.492962	-0.451041
H	-2.856689	-0.798184	-1.187419
H	-3.391876	-0.958102	0.491217
C	1.293349	-1.197407	-0.558285
C	1.640614	0.219417	-1.026600
C	1.115565	1.122660	0.128615
C	0.506655	0.103805	1.112540
C	-0.764843	-0.539241	0.537048
C	-0.230114	-1.367138	-0.662830
H	-0.611266	-0.974847	-1.607511
H	-0.526496	-2.413632	-0.583614
H	-1.165938	-1.230012	1.295369
C	1.490978	-1.065382	0.963429
H	1.186357	-1.953730	1.519979
H	2.512407	-0.798147	1.239564
H	0.372668	0.504937	2.117461
H	1.956257	1.612286	0.623119
H	2.716499	0.341093	-1.159206
H	1.150678	0.486819	-1.963204
H	1.859783	-1.987151	-1.049137

TABLE S32: Cartesian coordinates in Angstroms (Å) for the M06-2X/TZ optimized 2,6-disubstituted norbornane +HB gauche conformation with an OH donor and $NHCH_3$ acceptor group.

Atom	x	y	z
O	0.542094	2.028570	-0.298938
H	1.060264	1.366479	-0.784588
N	1.702689	-0.445977	-0.589065
H	1.978747	-1.109857	-1.301755
C	2.829842	-0.174180	0.296008
H	3.074393	-1.022755	0.947274
H	3.707448	0.077857	-0.296434
H	2.591495	0.684486	0.923370
C	-1.810578	-0.635857	-0.359837
C	-0.584921	-1.417566	-0.852913
C	0.538785	-0.975344	0.121337
C	-0.171221	0.041678	1.040627
C	-0.533817	1.332343	0.278402
C	-1.617730	0.842206	-0.727764
C	-1.555703	-0.611969	1.159009
H	-1.267625	0.977422	-1.751311
H	-2.543463	1.405779	-0.614551
H	-0.985262	2.026653	0.988630
H	-2.268776	0.005082	1.706159
H	-1.526263	-1.606568	1.605594
H	0.354808	0.228617	1.974939
H	0.839886	-1.827036	0.748613
H	-0.749103	-2.493284	-0.794033
H	-0.330351	-1.168797	-1.884376
H	-2.759872	-1.059064	-0.679491

TABLE S33: Cartesian coordinates in Angstroms (Å) for the df-MP2/TZ optimized 2,6-disubstituted norbornane +HB gauche conformation with an OH donor and $NHCH_3$ acceptor group.

Atom	x	y	z
O	0.536701	1.994479	-0.338716
H	1.058192	1.291806	-0.772989
N	1.707396	-0.442170	-0.586726
H	1.972770	-1.109465	-1.302120
C	2.850239	-0.201195	0.292396
H	3.095419	-1.067116	0.917873
H	3.720589	0.058005	-0.306238
H	2.625898	0.642567	0.941216
C	-1.800131	-0.680449	-0.331676
C	-0.566232	-1.443604	-0.828559
C	0.555570	-0.983435	0.137870
C	-0.159010	0.033946	1.048039
C	-0.536814	1.307241	0.267917
C	-1.631951	0.794225	-0.714093
C	-1.533001	-0.636156	1.183193
H	-1.300802	0.925486	-1.743930
H	-2.562520	1.347103	-0.586938
H	-0.981517	2.014444	0.969385
H	-2.249997	-0.021618	1.728670
H	-1.486113	-1.624991	1.641775
H	0.368483	0.239500	1.977493
H	0.869780	-1.827433	0.769395
H	-0.711941	-2.522001	-0.768235
H	-0.320903	-1.189304	-1.860959
H	-2.745247	-1.122628	-0.639287

TABLE S34: Cartesian coordinates in Angstroms (Å) for the df-MP2/aTZ optimized 2,6-disubstituted norbornane +HB gauche conformation with an OH donor and $NHCH_3$ acceptor group.

Atom	x	y	z
O	0.530125	1.995160	-0.358274
H	1.069215	1.290994	-0.772026
N	1.717961	-0.442735	-0.568411
H	1.984647	-1.107085	-1.287250
C	2.855661	-0.211564	0.321891
H	3.091771	-1.086216	0.939947
H	3.731369	0.051698	-0.268333
H	2.627127	0.625526	0.979434
C	-1.797835	-0.686042	-0.337775
C	-0.556782	-1.444525	-0.826527
C	0.556880	-0.977775	0.146624
C	-0.165038	0.044731	1.045637
C	-0.545531	1.310346	0.254014
C	-1.636190	0.787772	-0.728366
C	-1.537719	-0.630822	1.178885
H	-1.302559	0.915237	-1.758717
H	-2.570177	1.337306	-0.604627
H	-0.991653	2.025164	0.948099
H	-2.260266	-0.014740	1.716916
H	-1.487193	-1.617362	1.644007
H	0.357601	0.259941	1.976784
H	0.865475	-1.818718	0.786708
H	-0.697425	-2.524428	-0.766067
H	-0.306086	-1.188709	-1.858246
H	-2.739972	-1.135627	-0.647222

TABLE S35: Cartesian coordinates in Angstroms (Å) for the M06-2X/TZ optimized 2,6-disubstituted norbornane +HB conformation with an OH donor and $N(CH_3)_2$ acceptor group.

Atom	x	y	z
O	-0.196226	2.031266	-0.750753
H	0.527119	1.432513	-0.995545
N	1.631135	0.009575	-0.186678
C	2.317384	0.898506	0.744409
H	2.629214	0.367297	1.656691
H	3.206112	1.304896	0.263364
H	1.669387	1.727829	1.017821
C	2.565850	-0.995509	-0.671147
H	2.953925	-1.614518	0.153134
H	2.085344	-1.651171	-1.393423
H	3.407601	-0.506012	-1.158523
C	-1.751835	-0.654237	1.213551
C	-1.797218	-1.063663	-0.268737
C	-0.349741	-1.519425	-0.513387
C	0.481062	-0.631826	0.455259
C	-0.581019	0.323083	1.035580
C	-1.146057	1.263088	-0.052340
C	-1.983305	0.299128	-0.945154
H	-1.613277	0.320057	-1.969965
H	-3.032955	0.592342	-0.960962
H	-1.807525	1.984079	0.429465
H	-2.665787	-0.164065	1.549900
H	-1.519464	-1.480021	1.886783
H	-0.257753	0.844397	1.933843
H	0.851932	-1.253701	1.288500
H	-0.212756	-2.577686	-0.292766
H	-0.055732	-1.350865	-1.549736
H	-2.543937	-1.806318	-0.539318

TABLE S36: Cartesian coordinates in Angstroms (Å) for the df-MP2/TZ optimized 2,6-disubstituted norbornane +HB conformation with an OH donor and $N(CH_3)_2$ acceptor group.

Atom	x	y	z
O	-0.157080	1.978063	-0.779167
H	0.574562	1.353790	-0.950957
N	1.641362	0.007201	-0.180756
C	2.343020	0.871505	0.766300
H	2.662141	0.316305	1.660176
H	3.226424	1.284695	0.282697
H	1.700464	1.694671	1.064398
C	2.574273	-1.000991	-0.670600
H	2.953893	-1.629067	0.149420
H	2.092714	-1.643892	-1.401831
H	3.419500	-0.508807	-1.147565
C	-1.727009	-0.668162	1.231552
C	-1.770555	-1.097846	-0.243549
C	-0.321342	-1.544748	-0.484269
C	0.503133	-0.643653	0.475446
C	-0.562053	0.312222	1.041936
C	-1.120626	1.231270	-0.064547
C	-1.962913	0.253020	-0.936404
H	-1.598901	0.260372	-1.963187
H	-3.013227	0.544930	-0.946691
H	-1.778449	1.967121	0.399328
H	-2.643802	-0.177817	1.560891
H	-1.489527	-1.482906	1.917004
H	-0.244478	0.847988	1.933590
H	0.883343	-1.254168	1.313576
H	-0.176670	-2.599912	-0.253865
H	-0.030105	-1.381282	-1.522057
H	-2.512180	-1.849896	-0.503907

TABLE S37: Cartesian coordinates in Angstroms (Å) for the df-MP2/TZ optimized 2,6-disubstituted norbornane +HB conformation with an OH donor and $N(CH_3)_2$ acceptor group.

Atom	x	y	z
O	-0.160858	1.972173	-0.796696
H	0.581444	1.354142	-0.954677
N	1.645257	0.010781	-0.175194
C	2.347063	0.868142	0.779656
H	2.663497	0.301698	1.668553
H	3.231973	1.285058	0.300294
H	1.703591	1.688652	1.086809
C	2.576978	-1.001795	-0.661864
H	2.947261	-1.631333	0.162433
H	2.095259	-1.642446	-1.396323
H	3.427709	-0.511698	-1.133111
C	-1.726635	-0.665285	1.232434
C	-1.769370	-1.101017	-0.241843
C	-0.317768	-1.541899	-0.484304
C	0.502936	-0.637954	0.476394
C	-0.564043	0.318687	1.038585
C	-1.125636	1.230566	-0.072810
C	-1.968361	0.247276	-0.939063
H	-1.607353	0.252482	-1.967775
H	-3.020373	0.536370	-0.945143
H	-1.781387	1.972551	0.386178
H	-2.645639	-0.175651	1.559436
H	-1.484737	-1.476979	1.921292
H	-0.247798	0.859923	1.928600
H	0.881157	-1.247145	1.317959
H	-0.167613	-2.597522	-0.255597
H	-0.027355	-1.373576	-1.522595
H	-2.508955	-1.857530	-0.499192

TABLE S38: Cartesian coordinates in Angstroms (Å) for the M06-2X/TZ optimized 2,6-disubstituted norbornane +HB conformation with an OH donor and PH_2 acceptor group.

Atom	x	y	z
O	0.289574	2.207044	-0.072518
H	0.870785	1.831601	-0.741665
P	2.239674	-0.139702	-0.161371
H	2.813038	0.189056	1.088821
H	2.936912	-1.364221	-0.289363
C	-1.675901	-0.724372	0.954697
C	-1.561563	-0.842320	-0.576301
C	-0.183868	-1.503964	-0.721278
C	0.633876	-0.869332	0.445660
C	-0.379773	0.089917	1.107687
C	-0.742963	1.296991	0.223507
C	-1.432345	0.631940	-0.999015
H	-0.821112	0.746903	-1.896603
H	-2.400942	1.086174	-1.202589
H	-1.475323	1.887330	0.775551
H	-2.561401	-0.177420	1.279860
H	-1.642259	-1.688653	1.461451
H	-0.098700	0.378220	2.118573
H	0.875537	-1.637846	1.177685
H	-0.247026	-2.585007	-0.598790
H	0.254741	-1.309110	-1.702104
H	-2.361422	-1.382254	-1.077030

TABLE S39: Cartesian coordinates in Angstroms (Å) for the df-MP2/TZ optimized 2,6-disubstituted norbornane +HB conformation with an OH donor and PH_2 acceptor group.

Atom	x	y	z
O	0.254518	2.190693	-0.055386
H	0.882639	1.763903	-0.652579
P	2.186825	-0.169087	-0.148577
H	2.793206	0.110667	1.096107
H	2.868712	-1.391037	-0.344172
C	-1.724891	-0.748909	0.942616
C	-1.597255	-0.865690	-0.586145
C	-0.221119	-1.528669	-0.720644
C	0.584980	-0.901395	0.457942
C	-0.428787	0.060748	1.110696
C	-0.779583	1.268411	0.226066
C	-1.454129	0.606157	-1.005517
H	-0.827163	0.716978	-1.891724
H	-2.416753	1.066846	-1.223037
H	-1.519473	1.855871	0.770469
H	-2.611524	-0.198437	1.259896
H	-1.698444	-1.713966	1.449211
H	-0.159196	0.344765	2.126349
H	0.817369	-1.673741	1.190360
H	-0.283727	-2.610854	-0.605699
H	0.226915	-1.323839	-1.695427
H	-2.394526	-1.402811	-1.095043

TABLE S40: Cartesian coordinates in Angstroms (Å) for the df-MP2/aTZ optimized 2,6-disubstituted norbornane +HB conformation with an OH donor and PH_2 acceptor group.

Atom	x	y	z
O	0.251672	2.193884	-0.057496
H	0.899455	1.763395	-0.633522
P	2.189586	-0.172227	-0.142853
H	2.804673	0.085893	1.103206
H	2.861061	-1.397250	-0.360205
C	-1.725593	-0.750594	0.940523
C	-1.596812	-0.865428	-0.589240
C	-0.218227	-1.524676	-0.724628
C	0.584763	-0.900988	0.458459
C	-0.429806	0.061201	1.110389
C	-0.782933	1.268685	0.225773
C	-1.457074	0.607701	-1.007387
H	-0.829689	0.722478	-1.893721
H	-2.421676	1.067662	-1.222070
H	-1.521544	1.858347	0.771197
H	-2.613783	-0.201028	1.257957
H	-1.696180	-1.716953	1.446397
H	-0.161031	0.345990	2.127046
H	0.814268	-1.675740	1.190841
H	-0.278130	-2.608399	-0.615020
H	0.232229	-1.312029	-1.697643
H	-2.392972	-1.404651	-1.099732

TABLE S41: Cartesian coordinates in Angstroms (Å) for the M06-2X/TZ optimized 2,6-disubstituted norbornane +HB anti conformation with an OH donor and $PHCH_3$ acceptor group.

Atom	x	y	z
O	-0.781197	2.248114	-0.158742
H	-0.032880	2.042708	-0.730521
P	1.831562	0.647105	-0.040176
H	2.162048	1.209655	1.215586
C	3.229850	-0.556495	-0.167251
H	3.339352	-1.159159	0.733284
H	3.044553	-1.216119	-1.014408
H	4.158038	-0.020654	-0.356903
C	-1.751531	-1.123099	0.941087
C	-1.520841	-1.251259	-0.575832
C	0.002879	-1.431514	-0.631873
C	0.506069	-0.523406	0.533303
C	-0.798696	0.073501	1.104935
C	-1.480463	1.066007	0.145068
C	-1.853161	0.168614	-1.067529
H	-1.265992	0.445702	-1.945181
H	-2.904972	0.274618	-1.329494
H	-2.392473	1.408794	0.635327
H	-2.783117	-0.883345	1.200906
H	-1.435900	-2.005386	1.497808
H	-0.682153	0.472792	2.110401
H	0.946438	-1.148449	1.312320
H	0.287507	-2.469718	-0.461173
H	0.405253	-1.134972	-1.603412
H	-2.073638	-2.040029	-1.080322

TABLE S42: Cartesian coordinates in Angstroms (Å) for the df-MP2/TZ optimized 2,6-disubstituted norbornane +HB anti conformation with an OH donor and $PHCH_3$ acceptor group.

Atom	x	y	z
O	-0.766144	2.213907	-0.170797
H	0.024497	1.968730	-0.672337
P	1.816747	0.594096	-0.024894
H	2.161557	1.155270	1.226004
C	3.208828	-0.612844	-0.166498
H	3.310732	-1.229684	0.724827
H	3.027759	-1.257051	-1.025161
H	4.140205	-0.077680	-0.341229
C	-1.772838	-1.151441	0.932678
C	-1.524426	-1.290322	-0.579076
C	-0.002794	-1.478100	-0.616883
C	0.490419	-0.569312	0.551834
C	-0.815134	0.038845	1.103625
C	-1.475236	1.029421	0.130051
C	-1.836043	0.127848	-1.082291
H	-1.230907	0.394702	-1.949934
H	-2.882720	0.242779	-1.361103
H	-2.392200	1.379814	0.604931
H	-2.806272	-0.902302	1.177634
H	-1.469567	-2.032346	1.499190
H	-0.710275	0.440803	2.109756
H	0.920185	-1.193644	1.338891
H	0.277349	-2.516976	-0.441341
H	0.409702	-1.181167	-1.584152
H	-2.075936	-2.079131	-1.086049

TABLE S43: Cartesian coordinates in Angstroms (Å) for the df-MP2/aTZ optimized 2,6-disubstituted norbornane +HB anti conformation with an OH donor and $PHCH_3$ acceptor group.

Atom	x	y	z
O	-0.760299	2.219812	-0.170727
H	0.041628	1.975945	-0.658116
P	1.822710	0.585718	-0.026018
H	2.185487	1.136444	1.225526
C	3.198660	-0.637157	-0.189552
H	3.298296	-1.259114	0.699435
H	2.998575	-1.273994	-1.050389
H	4.134891	-0.110640	-0.369215
C	-1.773726	-1.144139	0.939239
C	-1.529578	-1.283497	-0.574140
C	-0.007759	-1.473430	-0.616758
C	0.490221	-0.568365	0.553395
C	-0.812043	0.044639	1.107745
C	-1.473317	1.035775	0.135049
C	-1.840530	0.135629	-1.077036
H	-1.237316	0.402986	-1.946844
H	-2.889190	0.253166	-1.350666
H	-2.387196	1.392651	0.612972
H	-2.806700	-0.892103	1.186932
H	-1.468947	-2.025964	1.505223
H	-0.703570	0.447642	2.114101
H	0.919012	-1.195942	1.340022
H	0.271685	-2.513970	-0.444825
H	0.403647	-1.171887	-1.584153
H	-2.083968	-2.072216	-1.080219

TABLE S44: Cartesian coordinates in Angstroms (Å) for the M06-2X/TZ optimized 2,6-disubstituted norbornane +HB gauche conformation with an OH donor and $PHCH_3$ acceptor group.

Atom	x	y	z
O	0.192246	2.123313	-0.361369
H	0.590669	1.650599	-1.100748
P	1.823108	-0.408120	-0.656979
H	2.369128	-1.704738	-0.817406
C	2.894938	0.173809	0.729957
H	2.881801	-0.507406	1.579850
H	3.918791	0.290463	0.379433
H	2.526860	1.150172	1.042861
C	-1.837382	-0.510053	1.207180
C	-2.027609	-0.718656	-0.306078
C	-0.781684	-1.547993	-0.646954
C	0.309013	-0.939601	0.286198
C	-0.458587	0.156209	1.061771
C	-0.856339	1.351507	0.177774
C	-1.822323	0.706164	-0.852848
H	-1.374050	0.698940	-1.848592
H	-2.758267	1.259017	-0.918580
H	-1.410453	2.047798	0.808545
H	-2.583022	0.151095	1.650030
H	-1.808025	-1.445195	1.766198
H	0.035369	0.457954	1.983450
H	0.627648	-1.686079	1.015108
H	-0.935562	-2.602766	-0.419912
H	-0.522664	-1.472025	-1.704798
H	-2.961279	-1.183816	-0.612765

TABLE S45: Cartesian coordinates in Angstroms (Å) for the df-MP2/TZ optimized 2,6-disubstituted norbornane +HB gauche conformation with an OH donor and $PHCH_3$ acceptor group.

Atom	x	y	z
O	0.173862	2.114225	-0.370354
H	0.615374	1.576390	-1.043139
P	1.787168	-0.421432	-0.631946
H	2.306743	-1.720604	-0.837895
C	2.903024	0.113264	0.737689
H	2.903384	-0.586660	1.571554
H	3.918736	0.222602	0.362138
H	2.561921	1.087526	1.082794
C	-1.858057	-0.501308	1.237913
C	-2.047699	-0.735374	-0.270346
C	-0.801940	-1.566845	-0.598518
C	0.286057	-0.945124	0.328883
C	-0.479918	0.162438	1.084527
C	-0.875434	1.340786	0.180611
C	-1.838408	0.677321	-0.840566
H	-1.384996	0.651032	-1.832742
H	-2.772957	1.231181	-0.918938
H	-1.432258	2.046140	0.798418
H	-2.603952	0.168286	1.668510
H	-1.828387	-1.426948	1.813319
H	0.010231	0.478271	2.003764
H	0.605219	-1.681971	1.068992
H	-0.951677	-2.619950	-0.359681
H	-0.541390	-1.498645	-1.656751
H	-2.981396	-1.206401	-0.569780

TABLE S46: Cartesian coordinates in Angstroms (Å) for the df-MP2/aTZ optimized 2,6-disubstituted norbornane +HB gauche conformation with an OH donor and $PHCH_3$ acceptor group.

Atom	x	y	z
O	0.176809	2.117488	-0.388709
H	0.621564	1.569673	-1.053695
P	1.778218	-0.434106	-0.653321
H	2.267073	-1.741710	-0.886496
C	2.925613	0.058090	0.706628
H	2.926288	-0.662011	1.524194
H	3.936703	0.159330	0.314650
H	2.604203	1.029905	1.078984
C	-1.842233	-0.481449	1.267514
C	-2.058428	-0.719420	-0.237567
C	-0.820698	-1.556098	-0.585824
C	0.284135	-0.940473	0.325977
C	-0.462754	0.175350	1.088723
C	-0.868579	1.352064	0.186349
C	-1.853798	0.691186	-0.815930
H	-1.417583	0.661596	-1.816550
H	-2.787630	1.250182	-0.876876
H	-1.407975	2.066208	0.810990
H	-2.578060	0.194049	1.708287
H	-1.805744	-1.406362	1.845400
H	0.045075	0.493627	1.998633
H	0.607702	-1.678443	1.064902
H	-0.970997	-2.609860	-0.346416
H	-0.574416	-1.485460	-1.648289
H	-2.999445	-1.188679	-0.519968

TABLE S47: Cartesian coordinates in Angstroms (Å) for the M06-2X/TZ optimized 2,6-disubstituted norbornane +HB conformation with an OH donor and OCH_3 acceptor group.

Atom	x	y	z
O	-0.628434	2.148429	-0.675785
H	-0.017960	1.689155	-1.265129
P	1.654600	0.145236	-0.454658
C	2.383343	1.203581	0.866180
H	2.487985	0.657505	1.805599
H	3.363454	1.559327	0.550851
H	1.736440	2.066919	1.016819
C	2.815527	-1.287741	-0.400007
H	2.964874	-1.640136	0.622168
H	2.416044	-2.102894	-1.002695
H	3.776711	-0.996369	-0.821228
C	-2.007553	-0.677528	1.229234
C	-2.000431	-1.154847	-0.234233
C	-0.551411	-1.640634	-0.378913
C	0.248229	-0.618598	0.486105
C	-0.852176	0.318988	1.033641
C	-1.475157	1.208882	-0.056921
C	-2.141601	0.171875	-1.002456
H	-1.626063	0.143080	-1.964381
H	-3.183379	0.424154	-1.195268
H	-2.247230	1.812413	0.422000
H	-2.940297	-0.193956	1.521632
H	-1.776154	-1.472208	1.938412
H	-0.541126	0.881363	1.911941
H	0.693506	-1.132572	1.343083
H	-0.432725	-2.651937	0.010202
H	-0.232529	-1.648603	-1.423567
H	-2.742417	-1.904597	-0.498177

TABLE S48: Cartesian coordinates in Angstroms (Å) for the df-MP2/TZ optimized 2,6-disubstituted norbornane +HB conformation with an OH donor and OCH_3 acceptor group.

Atom	x	y	z
O	-0.592308	2.131413	-0.667887
H	0.052674	1.628822	-1.188592
P	1.642676	0.100929	-0.437483
C	2.429493	1.114101	0.883125
H	2.545577	0.545852	1.807463
H	3.409744	1.452669	0.549826
H	1.811154	1.990341	1.069551
C	2.767875	-1.358659	-0.431899
H	2.915323	-1.739384	0.580146
H	2.347895	-2.148972	-1.052183
H	3.733119	-1.078717	-0.851450
C	-2.012204	-0.664182	1.262551
C	-2.015173	-1.152543	-0.195764
C	-0.574309	-1.656107	-0.343172
C	0.239889	-0.641713	0.517465
C	-0.844026	0.314142	1.059705
C	-1.452510	1.203048	-0.037086
C	-2.135839	0.168479	-0.972617
H	-1.620298	0.124364	-1.933096
H	-3.173741	0.436799	-1.165761
H	-2.214967	1.822312	0.436779
H	-2.937741	-0.164263	1.552054
H	-1.790197	-1.456337	1.978221
H	-0.526121	0.877060	1.935485
H	0.681885	-1.158817	1.376050
H	-0.464147	-2.668304	0.046940
H	-0.260249	-1.665266	-1.389373
H	-2.767845	-1.894991	-0.452277

TABLE S49: Cartesian coordinates in Angstroms (Å) for the df-MP2/aTZ optimized 2,6-disubstituted norbornane +HB conformation with an OH donor and OCH_3 acceptor group.

Atom	x	y	z
O	-0.621481	2.119882	-0.697001
H	0.048833	1.626909	-1.197543
P	1.648179	0.125437	-0.417531
C	2.418792	1.136641	0.914484
H	2.538095	0.555216	1.831240
H	3.396082	1.490422	0.586087
H	1.787473	2.002474	1.110582
C	2.780319	-1.328837	-0.408034
H	2.916282	-1.711169	0.605967
H	2.368383	-2.117810	-1.036618
H	3.748842	-1.039737	-0.815621
C	-2.014193	-0.681890	1.248611
C	-1.995370	-1.182864	-0.206378
C	-0.543972	-1.661960	-0.337575
C	0.244143	-0.628571	0.525069
C	-0.859996	0.314795	1.047806
C	-1.473980	1.182965	-0.063038
C	-2.132251	0.129522	-0.996252
H	-1.606861	0.087508	-1.952388
H	-3.173774	0.379541	-1.199146
H	-2.249408	1.797415	0.397841
H	-2.951379	-0.194566	1.525045
H	-1.783683	-1.464651	1.973219
H	-0.559193	0.892203	1.921432
H	0.685366	-1.132491	1.393487
H	-0.419341	-2.670682	0.059475
H	-0.218435	-1.669289	-1.381300
H	-2.733007	-1.941507	-0.463381

III. −HB CONFORMATIONS

TABLE S50: Cartesian coordinates in Angstroms (Å) for the M06-2X/TZ optimized 2,6-disubstituted norbornane −HB conformation with an OH donor and F acceptor group.

Atom	x	y	z
O	-1.896265	-1.004602	-0.133502
H	-2.195458	-1.821668	-0.538455
F	-1.382043	1.643391	-0.189110
C	1.462476	-0.222012	1.015307
C	1.506044	-0.263320	-0.526595
C	0.874971	1.091270	-0.883039
C	-0.124436	1.322334	0.270801
C	-0.054521	0.030295	1.088629
C	-0.570120	-1.186376	0.309933
C	0.478482	-1.363298	-0.824541
H	0.005533	-1.235733	-1.798376
H	0.934442	-2.352885	-0.789961
H	-0.525294	-2.046037	0.987524
H	1.759482	-1.161182	1.481797
H	2.052314	0.589511	1.441948
H	-0.492923	0.122039	2.079178
H	0.190780	2.155261	0.902826
H	1.619291	1.885227	-0.924982
H	0.341407	1.072077	-1.832525
H	2.482786	-0.430341	-0.973344

TABLE S51: Cartesian coordinates in Angstroms (Å) for the df-MP2/TZ optimized 2,6-disubstituted norbornane −HB conformation with an OH donor and F acceptor group.

Atom	x	y	z
O	-1.843039	-1.045331	-0.130216
H	-2.134076	-1.884772	-0.498777
F	-1.322953	1.626942	-0.173320
C	1.519261	-0.260199	1.011861
C	1.563096	-0.303242	-0.528774
C	0.926489	1.046284	-0.887390
C	-0.061839	1.283645	0.273160
C	0.002882	-0.008811	1.086094
C	-0.509004	-1.226018	0.311383
C	0.537445	-1.403055	-0.823442
H	0.062138	-1.274849	-1.795676
H	0.993204	-2.392726	-0.788471
H	-0.458746	-2.083011	0.991096
H	1.816638	-1.198985	1.479227
H	2.108113	0.552419	1.438224
H	-0.432188	0.082267	2.078144
H	0.272867	2.106710	0.907143
H	1.667504	1.842208	-0.943092
H	0.384268	1.015819	-1.831229
H	2.540197	-0.468675	-0.976156

TABLE S52: Cartesian coordinates in Angstroms (Å) for the df-MP2/aTZ optimized 2,6-disubstituted norbornane −HB conformation with an OH donor and F acceptor group.

Atom	x	y	z
O	-1.846642	-1.050239	-0.124731
H	-2.148315	-1.894388	-0.477440
F	-1.325591	1.619856	-0.188432
C	1.519314	-0.259105	1.015503
C	1.565228	-0.302156	-0.525863
C	0.934479	1.050698	-0.884123
C	-0.060879	1.284888	0.271681
C	0.001573	-0.006147	1.088139
C	-0.507571	-1.225282	0.314323
C	0.535901	-1.398951	-0.824081
H	0.059275	-1.265936	-1.795863
H	0.990081	-2.390365	-0.792946
H	-0.456584	-2.082582	0.994265
H	1.813837	-1.199118	1.484288
H	2.108016	0.554404	1.442568
H	-0.435086	0.087105	2.080333
H	0.259158	2.114538	0.905646
H	1.678661	1.845568	-0.928579
H	0.398823	1.026820	-1.832869
H	2.543290	-0.470822	-0.972222

TABLE S53: Cartesian coordinates in Angstroms (Å) for the M06-2X/TZ optimized 2,6-disubstituted norbornane −HB conformation with an OH donor and Cl acceptor group.

Atom	x	y	z
O	0.268409	2.081763	-0.107856
H	-0.102314	2.849480	-0.549013
Cl	2.287328	-0.123060	-0.086702
C	-1.528022	-0.882514	0.980148
C	-1.527897	-0.888429	-0.562042
C	-0.097913	-1.359588	-0.881520
C	0.705861	-0.847488	0.335785
C	-0.291794	0.031636	1.098180
C	-0.788924	1.246771	0.303342
C	-1.607333	0.614693	-0.858178
H	-1.161692	0.873019	-1.818896
H	-2.638971	0.966781	-0.850672
H	-1.461296	1.805327	0.963655
H	-2.426921	-0.435914	1.405315
H	-1.375248	-1.867021	1.421483
H	0.043987	0.282802	2.100984
H	0.969527	-1.677699	0.985489
H	-0.028693	-2.443904	-0.956517
H	0.276094	-0.931698	-1.809627
H	-2.304273	-1.481022	-1.039038

TABLE S54: Cartesian coordinates in Angstroms (Å) for the df-MP2/TZ optimized 2,6-disubstituted norbornane −HB conformation with an OH donor and Cl acceptor group.

Atom	x	y	z
O	0.194007	2.074911	-0.114048
H	-0.197939	2.852223	-0.523877
Cl	2.205118	-0.162862	-0.071932
C	-1.614471	-0.884810	0.973208
C	-1.615871	-0.890995	-0.567970
C	-0.186120	-1.355525	-0.891779
C	0.614264	-0.861433	0.333825
C	-0.376669	0.025717	1.091784
C	-0.871591	1.239604	0.298659
C	-1.693592	0.610741	-0.860752
H	-1.247885	0.868781	-1.821231
H	-2.724334	0.965678	-0.849844
H	-1.542312	1.797331	0.960807
H	-2.513008	-0.436220	1.397858
H	-1.462217	-1.869149	1.415935
H	-0.042868	0.274604	2.096214
H	0.851759	-1.701062	0.983742
H	-0.112782	-2.438078	-0.987131
H	0.187263	-0.906843	-1.810339
H	-2.393021	-1.483530	-1.044846

TABLE S55: Cartesian coordinates in Angstroms (Å) for the df-MP2/aTZ optimized 2,6-disubstituted norbornane −HB conformation with an OH donor and Cl acceptor group.

Atom	x	y	z
O	0.192034	2.078041	-0.108591
H	-0.197638	2.868173	-0.499111
Cl	2.198519	-0.147956	-0.082063
C	-1.612964	-0.889974	0.976237
C	-1.612425	-0.898762	-0.565741
C	-0.183059	-1.368030	-0.885107
C	0.617433	-0.862581	0.336549
C	-0.375216	0.022291	1.095008
C	-0.872944	1.234343	0.300821
C	-1.687091	0.603193	-0.863168
H	-1.235395	0.858985	-1.822316
H	-2.718474	0.958948	-0.858477
H	-1.546171	1.792154	0.960944
H	-2.512407	-0.440470	1.400297
H	-1.458584	-1.874484	1.420027
H	-0.041451	0.272480	2.100135
H	0.870226	-1.696357	0.989553
H	-0.111941	-2.452632	-0.967619
H	0.193703	-0.928887	-1.807913
H	-2.390666	-1.491221	-1.043148

TABLE S56: Cartesian coordinates in Angstroms (Å) for the M06-2X/TZ optimized 2,6-disubstituted norbornane −HB conformation with an OH donor and Br acceptor group.

Atom	x	y	z
O	-0.266042	2.092652	-0.093191
H	-0.642040	2.844254	-0.557157
Br	1.966205	-0.045929	-0.040955
C	-2.015663	-0.908959	0.963211
C	-1.985992	-0.915682	-0.578333
C	-0.538889	-1.352279	-0.873851
C	0.225378	-0.829896	0.363333
C	-0.800853	0.032495	1.104249
C	-1.312029	1.235617	0.299541
C	-2.095012	0.585124	-0.876157
H	-1.638980	0.853704	-1.829208
H	-3.134501	0.913100	-0.886542
H	-2.008024	1.779259	0.947612
H	-2.931653	-0.479861	1.369822
H	-1.851009	-1.889032	1.409738
H	-0.490729	0.290905	2.113329
H	0.491821	-1.654460	1.017090
H	-0.440902	-2.434199	-0.952081
H	-0.163401	-0.912479	-1.795504
H	-2.738853	-1.526553	-1.069729

TABLE S57: Cartesian coordinates in Angstroms (Å) for the df-MP2/TZ optimized 2,6-disubstituted norbornane −HB conformation with an OH donor and Br acceptor group.

Atom	x	y	z
O	-0.412628	2.089528	-0.112909
H	-0.805670	2.857983	-0.538263
Br	1.789413	-0.085059	-0.022176
C	-2.190479	-0.893749	0.950865
C	-2.164554	-0.897056	-0.589867
C	-0.717740	-1.324316	-0.895406
C	0.044605	-0.830739	0.353015
C	-0.971856	0.040416	1.092949
C	-1.474524	1.245453	0.290502
C	-2.273542	0.603249	-0.877862
H	-1.827403	0.875555	-1.834227
H	-3.312224	0.934438	-0.872318
H	-2.159253	1.798117	0.942299
H	-3.105378	-0.461782	1.357941
H	-2.027924	-1.874808	1.396939
H	-0.661356	0.294123	2.103602
H	0.275368	-1.671994	1.002334
H	-0.615239	-2.403244	-1.004987
H	-0.345129	-0.854428	-1.803623
H	-2.919270	-1.506429	-1.081418

TABLE S58: Cartesian coordinates in Angstroms (Å) for the df-MP2/aTZ optimized 2,6-disubstituted norbornane −HB conformation with an OH donor and Br acceptor group.

Atom	x	y	z
O	-0.419575	2.090216	-0.094827
H	-0.815525	2.870934	-0.498026
Br	1.778850	-0.062083	-0.036076
C	-2.182015	-0.911715	0.959222
C	-2.151351	-0.921052	-0.582317
C	-0.704861	-1.354782	-0.877339
C	0.055141	-0.837677	0.363093
C	-0.966319	0.027429	1.101465
C	-1.475676	1.227916	0.297314
C	-2.255264	0.578975	-0.880498
H	-1.793426	0.845807	-1.831793
H	-3.294446	0.910956	-0.892874
H	-2.171345	1.773571	0.944060
H	-3.099469	-0.480563	1.363709
H	-2.015195	-1.891742	1.408063
H	-0.656300	0.284968	2.112291
H	0.309273	-1.667744	1.019277
H	-0.604048	-2.436871	-0.963105
H	-0.325973	-0.902977	-1.793033
H	-2.906254	-1.531395	-1.074424

TABLE S59: Cartesian coordinates in Angstroms (Å) for the M06-2X/TZ optimized 2,6-disubstituted norbornane −HB conformation with an OH donor and OH acceptor group.

Atom	x	y	z
O	1.683240	1.375951	-0.141223
H	1.810780	2.239796	-0.539559
O	1.693860	-1.368733	-0.135257
H	1.826463	-2.232350	-0.532431
C	-1.477740	-0.005568	1.020920
C	-1.540721	-0.005396	-0.520593
C	-0.697002	-1.243829	-0.857116
C	0.352815	-1.280771	0.288882
C	0.060332	0.000329	1.079907
C	0.344063	1.282996	0.287922
C	-0.709330	1.241581	-0.854858
H	-0.211122	1.159184	-1.821074
H	-1.324099	2.142049	-0.859899
H	0.118704	2.121940	0.956352
H	-1.925975	0.880692	1.470311
H	-1.919172	-0.895226	1.470271
H	0.516753	0.002079	2.066901
H	0.128263	-2.120514	0.956627
H	-1.303452	-2.149833	-0.868823
H	-0.195982	-1.152214	-1.821056
H	-2.535548	-0.009707	-0.959591

TABLE S60: Cartesian coordinates in Angstroms (Å) for the df-MP2/TZ optimized 2,6-disubstituted norbornane −HB conformation with an OH donor and OH acceptor group.

Atom	x	y	z
O	1.648972	1.385155	-0.133741
H	1.761057	2.268295	-0.498386
O	1.658644	-1.373396	-0.135799
H	1.776900	-2.255178	-0.501779
C	-1.515769	-0.006085	1.012638
C	-1.580064	-0.005130	-0.527615
C	-0.736387	-1.241232	-0.864272
C	0.309407	-1.281252	0.283604
C	0.021577	-0.000739	1.072046
C	0.300425	1.282959	0.285557
C	-0.745101	1.237389	-0.862359
H	-0.239538	1.145596	-1.823501
H	-1.356284	2.140146	-0.877244
H	0.062231	2.116501	0.955563
H	-1.962927	0.880330	1.463197
H	-1.956695	-0.896300	1.461843
H	0.475152	0.000096	2.060424
H	0.077019	-2.117455	0.952331
H	-1.341209	-2.148237	-0.880598
H	-0.231457	-1.144379	-1.825251
H	-2.575217	-0.008289	-0.966780

TABLE S61: Cartesian coordinates in Angstroms (Å) for the df-MP2/aTZ optimized 2,6-disubstituted norbornane −HB conformation with an OH donor and OH acceptor group.

Atom	x	y	z
O	1.649915	1.384897	-0.134435
H	1.776159	2.276322	-0.477360
O	1.661316	-1.370922	-0.137189
H	1.794931	-2.260586	-0.481889
C	-1.518411	-0.007282	1.011647
C	-1.580407	-0.006014	-0.529448
C	-0.735717	-1.242438	-0.865286
C	0.308878	-1.281026	0.284251
C	0.020081	-0.000985	1.073835
C	0.298267	1.282969	0.286803
C	-0.745975	1.238016	-0.862825
H	-0.240242	1.149301	-1.825005
H	-1.358883	2.140659	-0.874491
H	0.065173	2.118392	0.956742
H	-1.966056	0.880042	1.462072
H	-1.958702	-0.899166	1.460309
H	0.471954	-0.000102	2.064011
H	0.082719	-2.119680	0.952528
H	-1.341137	-2.150094	-0.878738
H	-0.230750	-1.147640	-1.827288
H	-2.575724	-0.009691	-0.970584

TABLE S62: Cartesian coordinates in Angstroms (Å) for the M06-2X/TZ optimized 2,6-disubstituted norbornane −HB anti conformation with an OCH_3 donor and OH acceptor group.

Atom	x	y	z
C	1.225886	-1.219394	-0.592497
C	1.673574	0.195728	-0.986086
C	1.204994	1.063750	0.215466
C	0.458426	0.063375	1.106703
C	-0.822199	-0.482165	0.464003
C	-0.299799	-1.298860	-0.753716
H	-0.637235	-0.845410	-1.686362
H	-0.652989	-2.329606	-0.727442
H	-1.282255	-1.168904	1.188934
O	-1.754778	0.515463	0.148368
C	-3.002610	-0.015137	-0.210330
H	-3.667311	0.820361	-0.416372
H	-2.936295	-0.643403	-1.105467
H	-3.425642	-0.617864	0.602818
C	1.371102	-1.164953	0.941991
H	0.986354	-2.053008	1.443516
H	2.394298	-0.984062	1.271592
H	0.301228	0.430310	2.118086
H	2.080541	1.400247	0.782366
O	0.416281	2.179561	-0.130763
H	0.971411	2.803554	-0.603724
H	2.754642	0.250481	-1.116706
H	1.197611	0.539081	-1.904830
H	1.768339	-2.015221	-1.097219

TABLE S63: Cartesian coordinates in Angstroms (Å) for the df-MP2/TZ optimized 2,6-disubstituted norbornane −HB anti conformation with an OCH_3 donor and OH acceptor group.

Atom	x	y	z
O	0.425260	2.162783	-0.118853
H	1.014126	2.787910	-0.552546
O	-1.754770	0.493145	0.127914
C	-3.001960	-0.063361	-0.224447
H	-3.678647	0.762343	-0.423870
H	-2.932376	-0.688058	-1.119680
H	-3.408366	-0.672170	0.590771
C	1.225160	-1.249081	-0.602429
C	1.665128	0.167650	-0.993241
C	1.205658	1.029078	0.214696
C	0.450184	0.030613	1.095311
C	-0.824946	-0.514049	0.446388
C	-0.297778	-1.331361	-0.767912
C	1.363112	-1.196727	0.931555
H	-0.631814	-0.878508	-1.701729
H	-0.648712	-2.362869	-0.740477
H	-1.289020	-1.200316	1.169375
H	0.975421	-2.085753	1.429554
H	2.384587	-1.015648	1.267142
H	0.289255	0.392483	2.108036
H	2.085052	1.346372	0.786019
H	2.744421	0.228351	-1.135338
H	1.176062	0.512719	-1.904097
H	1.771876	-2.042851	-1.106703

TABLE S64: Cartesian coordinates in Angstroms (Å) for the df-MP2/aTZ optimized 2,6-disubstituted norbornane −HB anti conformation with an OCH_3 donor and OH acceptor group.

Atom	x	y	z
O	0.415132	2.167158	-0.120232
H	1.003290	2.811155	-0.529709
O	-1.757870	0.485479	0.123070
C	-3.003317	-0.080184	-0.233197
H	-3.681507	0.743403	-0.439960
H	-2.922107	-0.709624	-1.124939
H	-3.407475	-0.686503	0.585760
C	1.231630	-1.244062	-0.600147
C	1.666092	0.175035	-0.991223
C	1.201561	1.034194	0.216929
C	0.449080	0.033470	1.098074
C	-0.822603	-0.517915	0.447451
C	-0.291345	-1.333778	-0.766479
C	1.368473	-1.190686	0.934716
H	-0.628265	-0.882428	-1.701061
H	-0.637273	-2.367819	-0.737183
H	-1.287124	-1.203882	1.171627
H	0.982874	-2.081532	1.433018
H	2.389596	-1.003696	1.271057
H	0.285966	0.394639	2.111731
H	2.077331	1.360737	0.789197
H	2.746517	0.239930	-1.129309
H	1.178306	0.518357	-1.904328
H	1.782915	-2.035940	-1.104536

TABLE S65: Cartesian coordinates in Angstroms (Å) for the M06-2X/TZ optimized 2,6-disubstituted norbornane −HB gauche conformation with an OCH_3 donor and OH acceptor group.

Atom	x	y	z
O	0.653047	1.984656	-0.223076
H	0.373569	2.782944	-0.676512
O	1.653200	-0.620271	-0.599398
C	2.733977	-0.210012	0.199831
H	2.995984	-0.984021	0.931849
H	3.582214	-0.048108	-0.461478
H	2.509669	0.722536	0.722161
C	-1.560263	-0.567543	1.141105
C	-1.786527	-0.610320	-0.383194
C	-0.590903	-1.460952	-0.823883
C	0.532530	-1.018840	0.142505
C	-0.151638	0.043626	1.038122
C	-0.492372	1.329339	0.280928
C	-1.510547	0.849244	-0.787740
H	-1.067794	0.917210	-1.782025
H	-2.417519	1.453593	-0.771495
H	-0.999013	1.994396	0.989650
H	-2.263341	0.073825	1.673160
H	-1.564813	-1.557251	1.598065
H	0.363481	0.224857	1.979427
H	0.824965	-1.850312	0.799578
H	-0.801852	-2.524862	-0.720966
H	-0.285454	-1.269325	-1.851167
H	-2.750134	-0.989816	-0.714029

TABLE S66: Cartesian coordinates in Angstroms (Å) for the df-MP2/TZ optimized 2,6-disubstituted norbornane −HB gauche conformation with an OCH_3 donor and OH acceptor group.

Atom	x	y	z
O	0.641999	1.988646	-0.212867
H	0.346067	2.824359	-0.586039
O	1.641847	-0.632512	-0.598983
C	2.725026	-0.236816	0.217349
H	2.971321	-1.016524	0.946432
H	3.577835	-0.083783	-0.437985
H	2.508647	0.695288	0.740159
C	-1.566743	-0.568712	1.154888
C	-1.797084	-0.618233	-0.367278
C	-0.602534	-1.468495	-0.804812
C	0.521139	-1.021928	0.157798
C	-0.159745	0.044258	1.047326
C	-0.507170	1.324285	0.289178
C	-1.518459	0.836480	-0.780452
H	-1.067339	0.895669	-1.770976
H	-2.424679	1.442232	-0.775338
H	-1.021607	1.982950	0.997692
H	-2.268694	0.073965	1.687487
H	-1.567771	-1.557015	1.615497
H	0.353281	0.229265	1.988838
H	0.812938	-1.848949	0.820957
H	-0.810966	-2.532528	-0.698119
H	-0.297528	-1.277536	-1.832331
H	-2.761693	-0.999524	-0.694445

TABLE S67: Cartesian coordinates in Angstroms (Å) for the df-MP2/aTZ optimized 2,6-disubstituted norbornane −HB gauche conformation with an OCH_3 donor and OH acceptor group.

Atom	x	y	z
O	0.643764	1.988583	-0.217463
H	0.350446	2.821441	-0.602709
O	1.644808	-0.635433	-0.599644
C	2.726916	-0.238719	0.222237
H	2.970377	-1.022502	0.948869
H	3.580199	-0.080721	-0.432253
H	2.502972	0.690721	0.748367
C	-1.565370	-0.569430	1.156876
C	-1.799501	-0.616335	-0.365626
C	-0.604671	-1.464445	-0.809916
C	0.519572	-1.024169	0.155024
C	-0.157005	0.042846	1.047013
C	-0.506500	1.323825	0.290549
C	-1.524248	0.840355	-0.775742
H	-1.081547	0.904364	-1.770672
H	-2.431478	1.445930	-0.759167
H	-1.013580	1.986081	1.001717
H	-2.265669	0.074310	1.692128
H	-1.564896	-1.559347	1.616048
H	0.358829	0.225866	1.988499
H	0.810987	-1.853920	0.816205
H	-0.813413	-2.529859	-0.709437
H	-0.300608	-1.265824	-1.837286
H	-2.765460	-0.998131	-0.691416

TABLE S68: Cartesian coordinates in Angstroms (Å) for the M06-2X/TZ optimized 2,6-disubstituted norbornane −HB conformation with an SH donor and OH acceptor group.

Atom	x	y	z
O	0.377789	2.015824	-0.090132
H	0.099739	2.718462	-0.681748
S	2.279494	-0.093765	-0.082677
H	2.949523	-1.255912	-0.088548
C	-1.596492	-0.827601	0.971964
C	-1.581252	-0.832181	-0.568495
C	-0.174413	-1.373490	-0.870630
C	0.667492	-0.856548	0.328495
C	-0.324833	0.028866	1.103725
C	-0.749893	1.266142	0.306076
C	-1.583411	0.674402	-0.864626
H	-1.118131	0.907521	-1.823235
H	-2.596407	1.076832	-0.867466
H	-1.390713	1.880544	0.947156
H	-2.480915	-0.344218	1.388504
H	-1.489061	-1.818808	1.411735
H	0.013390	0.263169	2.110055
H	0.910218	-1.687300	0.987047
H	-0.161802	-2.462973	-0.906357
H	0.212079	-1.001991	-1.818247
H	-2.382799	-1.382545	-1.055073

TABLE S69: Cartesian coordinates in Angstroms (Å) for the df-MP2/TZ optimized 2,6-disubstituted norbornane −HB conformation with an SH donor and OH acceptor group.

Atom	x	y	z
O	0.341199	1.990207	-0.097569
H	0.042967	2.702137	-0.671959
S	2.229499	-0.130364	-0.073341
H	2.884137	-1.298468	-0.066979
C	-1.648547	-0.843233	0.963852
C	-1.635647	-0.845318	-0.575592
C	-0.228597	-1.378945	-0.883484
C	0.609819	-0.879276	0.323907
C	-0.376338	0.010702	1.097417
C	-0.797951	1.247077	0.301678
C	-1.637400	0.660138	-0.865830
H	-1.175174	0.894831	-1.825224
H	-2.649725	1.064548	-0.861508
H	-1.434333	1.863846	0.944431
H	-2.532952	-0.359659	1.381049
H	-1.540232	-1.834963	1.403127
H	-0.039710	0.241377	2.105487
H	0.835262	-1.717822	0.980991
H	-0.210669	-2.467703	-0.939232
H	0.158523	-0.986000	-1.822230
H	-2.437918	-1.395301	-1.062464

TABLE S70: Cartesian coordinates in Angstroms (Å) for the df-MP2/aTZ optimized 2,6-disubstituted norbornane −HB conformation with an SH donor and OH acceptor group.

Atom	x	y	z
O	0.355526	1.977334	-0.112189
H	0.063740	2.723842	-0.647173
S	2.232298	-0.142325	-0.061347
H	2.885716	-1.311968	-0.034501
C	-1.653087	-0.839882	0.960575
C	-1.640773	-0.835798	-0.579774
C	-0.231968	-1.364782	-0.893536
C	0.604525	-0.881033	0.321638
C	-0.378039	0.011020	1.097097
C	-0.793280	1.250342	0.301343
C	-1.646886	0.671544	-0.860746
H	-1.198061	0.915214	-1.825018
H	-2.660613	1.074312	-0.837964
H	-1.413315	1.881378	0.946777
H	-2.537174	-0.354980	1.379239
H	-1.544840	-1.833815	1.396971
H	-0.040207	0.238725	2.106436
H	0.822597	-1.726260	0.974236
H	-0.212359	-2.453394	-0.964163
H	0.157734	-0.956917	-1.825932
H	-2.443406	-1.384985	-1.069108

TABLE S71: Cartesian coordinates in Angstroms (Å) for the M06-2X/TZ optimized 2,6-disubstituted norbornane −HB anti conformation with an SCH_3 donor and OH acceptor group.

Atom	x	y	z
O	-0.611360	2.130505	-0.248702
H	-0.127413	2.546783	0.470392
S	1.849003	0.601961	-0.081292
C	3.233957	-0.561138	-0.021708
H	3.087576	-1.370993	-0.734502
H	4.129863	-0.010237	-0.298240
H	3.362374	-0.968769	0.979714
C	-1.681968	-1.137946	0.979403
C	-1.634587	-1.169401	-0.559834
C	-0.129776	-1.342028	-0.823094
C	0.522724	-0.568020	0.358397
C	-0.684226	0.026569	1.103037
C	-1.415189	1.092325	0.268357
C	-2.022425	0.277729	-0.897993
H	-1.599561	0.619317	-1.841855
H	-3.103757	0.400215	-0.942546
H	-2.212624	1.517794	0.886971
H	-2.670228	-0.893983	1.370801
H	-1.327003	-2.056341	1.447061
H	-0.438206	0.355511	2.111655
H	0.996130	-1.273324	1.042218
H	0.158118	-2.393401	-0.801637
H	0.168353	-0.927067	-1.784765
H	-2.255729	-1.919729	-1.042806

TABLE S72: Cartesian coordinates in Angstroms (Å) for the df-MP2/TZ optimized 2,6-disubstituted norbornane −HB anti conformation with an SCH_3 donor and OH acceptor group.

Atom	x	y	z
O	-0.649851	2.078741	-0.263336
H	-0.190301	2.514564	0.462863
S	1.829080	0.587281	-0.087708
C	3.221104	-0.566247	-0.017999
H	3.093592	-1.372963	-0.737020
H	4.117023	-0.007146	-0.278115
H	3.339631	-0.978326	0.982505
C	-1.663523	-1.197257	0.999836
C	-1.631797	-1.233255	-0.538478
C	-0.128783	-1.376669	-0.819503
C	0.522573	-0.597366	0.358249
C	-0.683650	-0.017585	1.111108
C	-1.436515	1.031938	0.280069
C	-2.045032	0.205055	-0.874825
H	-1.637871	0.549140	-1.824575
H	-3.128521	0.311658	-0.906067
H	-2.232032	1.450704	0.905226
H	-2.652309	-0.967750	1.399705
H	-1.288130	-2.107786	1.467845
H	-0.434084	0.316476	2.117399
H	1.006543	-1.299906	1.039990
H	0.180524	-2.422327	-0.809703
H	0.149156	-0.945734	-1.780328
H	-2.245872	-1.995420	-1.013012

TABLE S73: Cartesian coordinates in Angstroms (Å) for the df-MP2/aTZ optimized 2,6-disubstituted norbornane −HB anti conformation with an SCH_3 donor and OH acceptor group.

Atom	x	y	z
O	-0.649599	2.087459	-0.261107
H	-0.217672	2.545378	0.470130
S	1.822222	0.591168	-0.086702
C	3.203775	-0.577716	-0.072130
H	3.041977	-1.371619	-0.799578
H	4.098298	-0.023323	-0.349452
H	3.341068	-1.004731	0.920479
C	-1.668943	-1.187797	1.009349
C	-1.621699	-1.239619	-0.529091
C	-0.116377	-1.395675	-0.789869
C	0.523905	-0.593621	0.379463
C	-0.687953	-0.007524	1.118474
C	-1.437028	1.033120	0.273358
C	-2.022003	0.198306	-0.888217
H	-1.590317	0.531375	-1.832201
H	-3.104775	0.309461	-0.945670
H	-2.244229	1.449910	0.885900
H	-2.661548	-0.951987	1.398249
H	-1.297594	-2.094846	1.489169
H	-0.445750	0.336308	2.124293
H	1.012676	-1.281045	1.074880
H	0.187032	-2.443331	-0.751888
H	0.177245	-0.985989	-1.756497
H	-2.235287	-2.004146	-1.002660

TABLE S74: Cartesian coordinates in Angstroms (Å) for the M06-2X/TZ optimized 2,6-disubstituted norbornane −HB gauche conformation with an SCH_3 donor and OH acceptor group.

Atom	x	y	z
C	1.992114	-0.716905	-0.308562
C	1.745897	0.679482	-0.911740
C	0.831572	1.361793	0.127633
C	0.433635	0.195479	1.048383
C	-0.317439	-0.954632	0.339336
C	0.758462	-1.581390	-0.592720
H	0.465209	-1.543056	-1.641815
H	0.931390	-2.624457	-0.329981
H	-0.595821	-1.671767	1.113256
S	-1.823013	-0.610197	-0.609220
C	-2.781075	0.262499	0.643443
H	-3.779679	0.416215	0.241670
H	-2.855170	-0.326280	1.557119
H	-2.332677	1.230372	0.859746
C	1.822162	-0.456583	1.200530
H	1.806342	-1.371953	1.791890
H	2.567030	0.223901	1.614014
H	-0.047978	0.514743	1.972134
H	1.430651	2.048834	0.735180
O	-0.199563	2.089940	-0.503508
H	-0.561422	2.719846	0.122775
H	2.669547	1.238029	-1.054843
H	1.228245	0.628515	-1.869697
H	2.929750	-1.175099	-0.612811

TABLE S75: Cartesian coordinates in Angstroms (Å) for the df-MP2/TZ optimized 2,6-disubstituted norbornane −HB gauche conformation with an SCH_3 donor and OH acceptor group.

Atom	x	y	z
O	-0.166728	2.113468	-0.464918
H	-0.489547	2.751870	0.177373
S	-1.786873	-0.606290	-0.591732
C	-2.766965	0.246009	0.657482
H	-3.761802	0.394025	0.243778
H	-2.850162	-0.352050	1.563767
H	-2.332656	1.215847	0.887769
C	2.010292	-0.717566	-0.277693
C	1.754016	0.673524	-0.885676
C	0.859005	1.362712	0.162407
C	0.455268	0.199272	1.080087
C	-0.296150	-0.949562	0.373840
C	0.778495	-1.582850	-0.556050
H	0.482959	-1.545157	-1.604811
H	0.949763	-2.625403	-0.288937
H	-0.577030	-1.664665	1.150533
C	1.842421	-0.454658	1.229999
H	1.825376	-1.369980	1.822143
H	2.588216	0.226081	1.642549
H	-0.021934	0.518518	2.006247
H	1.473984	2.034990	0.770554
H	2.673801	1.232271	-1.050801
H	1.218042	0.612590	-1.832619
H	2.948920	-1.173906	-0.583439

TABLE S76: Cartesian coordinates in Angstroms (Å) for the df-MP2/aTZ optimized 2,6-disubstituted norbornane −HB gauche conformation with an SCH_3 donor and OH acceptor group.

Atom	x	y	z
O	-0.175024	2.112105	-0.458874
H	-0.458177	2.791731	0.161177
S	-1.781835	-0.597387	-0.597493
C	-2.765821	0.237856	0.661059
H	-3.758908	0.394753	0.244634
H	-2.850431	-0.379090	1.555298
H	-2.325770	1.201776	0.908569
C	2.010248	-0.718942	-0.277465
C	1.751629	0.672064	-0.886978
C	0.858137	1.360543	0.163821
C	0.454230	0.197343	1.081745
C	-0.296975	-0.950245	0.372841
C	0.778688	-1.585805	-0.554642
H	0.481870	-1.550560	-1.604112
H	0.950809	-2.628083	-0.283542
H	-0.586740	-1.665149	1.147866
C	1.842335	-0.456396	1.231308
H	1.824006	-1.373415	1.822394
H	2.588424	0.224824	1.644935
H	-0.024388	0.517710	2.007762
H	1.471590	2.036579	0.769852
H	2.671304	1.232294	-1.053771
H	1.212722	0.609457	-1.833288
H	2.950033	-1.174928	-0.583678

TABLE S77: Cartesian coordinates in Angstroms (Å) for the M06-2X/TZ optimized 2,6-disubstituted norbornane −HB anti conformation with an $NHCH_3$ donor and OH acceptor group.

Atom	x	y	z
C	1.240634	-1.221014	-0.597519
C	1.433411	0.218385	-1.127295
C	1.168953	1.092358	0.115507
C	0.522476	0.094489	1.087682
C	-0.775584	-0.522431	0.518656
C	-0.259394	-1.521264	-0.560208
H	-0.753112	-1.337576	-1.513725
H	-0.436839	-2.556648	-0.266783
H	-1.256259	-1.082519	1.335255
N	-1.702855	0.434251	-0.057988
H	-1.641283	1.333601	0.396281
C	-3.061373	-0.062589	-0.108643
H	-3.718184	0.691899	-0.538620
H	-3.101265	-0.942207	-0.754798
H	-3.461027	-0.358820	0.872741
C	1.515895	-1.065970	0.912098
H	1.248171	-1.956229	1.480892
H	2.542021	-0.784732	1.150612
H	0.405943	0.486870	2.097793
H	2.126768	1.391000	0.553589
O	0.431127	2.261236	-0.196495
H	0.661093	2.942219	0.438051
H	2.431987	0.385326	-1.528236
H	0.705556	0.467381	-1.899081
H	1.834674	-1.968679	-1.117518

TABLE S78: Cartesian coordinates in Angstroms (Å) for the df-MP2/TZ optimized 2,6-disubstituted norbornane −HB anti conformation with an $NHCH_3$ donor and OH acceptor group.

Atom	x	y	z
O	0.417385	2.251787	-0.171638
H	0.688014	2.916072	0.468063
N	-1.699385	0.413522	-0.077969
H	-1.647741	1.302479	0.400146
C	-3.061813	-0.085352	-0.115390
H	-3.721052	0.669523	-0.539060
H	-3.106484	-0.963178	-0.760513
H	-3.452134	-0.380960	0.868677
H	2.375933	0.380098	-1.551940
H	-0.437638	-2.587812	-0.239576
C	1.220246	-1.239335	-0.600437
C	1.389093	0.203103	-1.127100
C	1.149271	1.067434	0.124837
C	0.507600	0.066511	1.093138
C	-0.788799	-0.547832	0.522270
C	-0.274295	-1.554045	-0.547577
H	-0.778735	-1.383764	-1.498003
H	-1.281767	-1.096473	1.339624
C	1.505251	-1.087477	0.906520
H	1.246356	-1.982086	1.473018
H	2.531060	-0.799574	1.140042
H	0.394830	0.451743	2.106443
H	2.115385	1.349622	0.555153
H	0.638840	0.448571	-1.877627
H	1.817531	-1.979450	-1.128341

TABLE S79: Cartesian coordinates in Angstroms (Å) for the df-MP2/TZ optimized 2,6-disubstituted norbornane −HB anti conformation with an $NHCH_3$ donor and OH acceptor group.

Atom	x	y	z
O	0.401649	2.250406	-0.174945
H	0.737286	2.952468	0.390704
N	-1.707894	0.410574	-0.066350
H	-1.660917	1.301049	0.411770
C	-3.066613	-0.102606	-0.104391
H	-3.734854	0.651452	-0.517104
H	-3.102736	-0.975224	-0.758462
H	-3.446153	-0.411505	0.880515
H	2.394681	0.378299	-1.542206
H	-0.438932	-2.583210	-0.252562
C	1.225238	-1.237632	-0.601718
C	1.401148	0.203684	-1.129540
C	1.149225	1.070363	0.120202
C	0.509813	0.071066	1.091078
C	-0.785675	-0.546800	0.522943
C	-0.271286	-1.547183	-0.553382
H	-0.773242	-1.368124	-1.504620
H	-1.271337	-1.102114	1.341653
C	1.507959	-1.084193	0.906384
H	1.246116	-1.978096	1.474323
H	2.534498	-0.796062	1.140771
H	0.397336	0.460618	2.103564
H	2.110174	1.366674	0.553050
H	0.660091	0.448807	-1.890556
H	1.821474	-1.981312	-1.127932

TABLE S80: Cartesian coordinates in Angstroms (Å) for the M06-2X/TZ optimized 2,6-disubstituted norbornane −HB gauche conformation with an $NHCH_3$ donor and OH acceptor group.

Atom	x	y	z
O	0.658421	2.022478	-0.228383
H	0.374479	2.780928	-0.743177
N	1.664645	-0.632086	-0.587722
H	1.957545	-1.418387	-1.154638
C	2.772420	-0.184013	0.237892
H	3.020383	-0.882572	1.051241
H	3.657288	-0.049628	-0.382379
H	2.523073	0.782178	0.673697
C	-1.807576	-0.595658	-0.368020
C	-0.644771	-1.516281	-0.749527
C	0.501972	-1.035571	0.180294
C	-0.163393	0.074558	1.030782
C	-0.486646	1.344295	0.240067
C	-1.463961	0.829791	-0.847773
C	-1.589632	-0.492391	1.154356
H	-0.963671	0.817342	-1.816936
H	-2.348966	1.461778	-0.921753
H	-1.031498	2.007177	0.922758
H	-2.276846	0.189941	1.654690
H	-1.624198	-1.461982	1.650982
H	0.355881	0.275766	1.965925
H	0.760872	-1.838137	0.890576
H	-0.894900	-2.560426	-0.560491
H	-0.359769	-1.412741	-1.796639
H	-2.782891	-0.951923	-0.691050

TABLE S81: Cartesian coordinates in Angstroms (Å) for the df-MP2/TZ optimized 2,6-disubstituted norbornane −HB gauche conformation with an $NHCH_3$ donor and OH acceptor group.

Atom	x	y	z
O	0.658530	2.016067	-0.226244
H	0.353136	2.839996	-0.617653
N	1.660992	-0.641378	-0.591368
H	1.953382	-1.441472	-1.141459
C	2.774844	-0.209585	0.240885
H	3.016661	-0.914714	1.048670
H	3.659865	-0.079903	-0.378903
H	2.533799	0.754727	0.680969
C	-1.803174	-0.617250	-0.361267
C	-0.640244	-1.536965	-0.737672
C	0.505664	-1.048860	0.188234
C	-0.158476	0.062486	1.032455
C	-0.488853	1.326397	0.240131
C	-1.457062	0.802762	-0.849483
C	-1.582965	-0.506797	1.158998
H	-0.946714	0.782507	-1.812412
H	-2.341353	1.434235	-0.936412
H	-1.042936	1.981098	0.922686
H	-2.269848	0.176939	1.658682
H	-1.614001	-1.474831	1.659506
H	0.357494	0.268116	1.968384
H	0.768554	-1.847035	0.902476
H	-0.887153	-2.581069	-0.543343
H	-0.354040	-1.434739	-1.784605
H	-2.778952	-0.976015	-0.681630

TABLE S82: Cartesian coordinates in Angstroms (Å) for the df-MP2/aTZ optimized 2,6-disubstituted norbornane −HB gauche conformation with an $NHCH_3$ donor and OH acceptor group.

Atom	x	y	z
O	0.658094	2.015703	-0.235992
H	0.356317	2.841100	-0.630695
N	1.668143	-0.646009	-0.587161
H	1.961155	-1.442524	-1.143462
C	2.777385	-0.215647	0.253834
H	3.006984	-0.923475	1.063768
H	3.668126	-0.089746	-0.359575
H	2.533538	0.749740	0.692539
C	-1.805599	-0.616034	-0.360335
C	-0.640165	-1.530604	-0.745277
C	0.505514	-1.048883	0.184550
C	-0.155966	0.062747	1.030614
C	-0.490054	1.326871	0.239370
C	-1.467639	0.807400	-0.844999
C	-1.580534	-0.508319	1.160255
H	-0.969306	0.794565	-1.815305
H	-2.355179	1.437792	-0.916126
H	-1.035073	1.986233	0.925429
H	-2.267145	0.174839	1.663042
H	-1.607693	-1.478341	1.659037
H	0.362469	0.268014	1.966402
H	0.764102	-1.850211	0.898299
H	-0.885307	-2.577534	-0.559504
H	-0.354652	-1.418212	-1.792316
H	-2.782039	-0.977900	-0.678562

TABLE S83: Cartesian coordinates in Angstroms (Å) for the M06-2X/TZ optimized 2,6-disubstituted norbornane −HB conformation with an $N(CH_3)_2$ donor and OH acceptor group.

Atom	x	y	z
O	-0.363067	2.111325	-0.584278
H	-0.856797	2.652022	-1.204700
N	1.656428	0.037609	-0.227412
C	2.663308	-0.940599	-0.593720
H	2.242699	-1.695233	-1.256397
H	3.481215	-0.446145	-1.116530
H	3.079734	-1.455698	0.289910
C	2.248200	1.055467	0.620318
H	1.534528	1.854887	0.798752
H	2.584451	0.643080	1.587825
H	3.115325	1.481823	0.116461
C	-1.683990	-0.783008	1.191876
C	-1.706583	-1.168077	-0.298648
C	-0.243698	-1.569278	-0.526938
C	0.540960	-0.624624	0.426428
C	-0.568805	0.265840	1.027633
C	-1.250956	1.189848	0.012013
C	-1.932153	0.197899	-0.968494
H	-1.457388	0.255166	-1.948555
H	-2.992900	0.421282	-1.084536
H	-2.022794	1.743766	0.558702
H	-2.622477	-0.355607	1.545904
H	-1.398000	-1.608185	1.843646
H	-0.265476	0.781068	1.936627
H	0.930804	-1.218872	1.275327
H	-0.072462	-2.616780	-0.278611
H	0.060340	-1.408035	-1.560795
H	-2.426604	-1.929766	-0.588201

TABLE S84: Cartesian coordinates in Angstroms (Å) for the df-MP2/TZ optimized 2,6-disubstituted norbornane −HB conformation with an $N(CH_3)_2$ donor and OH acceptor group.

Atom	x	y	z
O	-0.347028	2.105690	-0.567215
H	-0.879000	2.693896	-1.111894
N	1.662278	0.027002	-0.239500
C	2.671558	-0.958673	-0.590236
H	2.254440	-1.711971	-1.254796
H	3.495360	-0.467407	-1.105264
H	3.075812	-1.471524	0.299371
C	2.264559	1.032098	0.620645
H	1.555461	1.831655	0.809533
H	2.603396	0.606967	1.580491
H	3.130584	1.458178	0.115989
C	-1.661192	-0.802050	1.197325
C	-1.683070	-1.190164	-0.291189
C	-0.222059	-1.594615	-0.512877
C	0.560752	-0.641353	0.432509
C	-0.547471	0.247503	1.032786
C	-1.233283	1.166967	0.020316
C	-1.898204	0.173035	-0.966951
H	-1.403889	0.227996	-1.936918
H	-2.956529	0.397087	-1.102745
H	-2.013996	1.706094	0.568632
H	-2.599553	-0.373759	1.551775
H	-1.373325	-1.625879	1.850512
H	-0.247281	0.762123	1.943086
H	0.961023	-1.227928	1.282936
H	-0.053004	-2.639799	-0.253307
H	0.084250	-1.440573	-1.547119
H	-2.404430	-1.951322	-0.580617

TABLE S85: Cartesian coordinates in Angstroms (Å) for the df-MP2/aTZ optimized 2,6-disubstituted norbornane −HB conformation with an $N(CH_3)_2$ donor and OH acceptor group.

Atom	x	y	z
O	-0.356711	2.109733	-0.566278
H	-0.883994	2.681994	-1.134573
N	1.664854	0.031785	-0.239828
C	2.676178	-0.954479	-0.588931
H	2.259488	-1.708112	-1.254857
H	3.502074	-0.461279	-1.100423
H	3.074849	-1.466296	0.304891
C	2.264374	1.035483	0.625647
H	1.551169	1.831471	0.820055
H	2.601959	0.602210	1.583343
H	3.130340	1.466478	0.123538
C	-1.657703	-0.808352	1.197234
C	-1.679753	-1.195147	-0.292585
C	-0.216865	-1.593282	-0.517346
C	0.562698	-0.638793	0.429800
C	-0.547454	0.246390	1.032751
C	-1.239237	1.163451	0.021766
C	-1.901922	0.168467	-0.966729
H	-1.411619	0.225916	-1.939584
H	-2.962482	0.389059	-1.097320
H	-2.020686	1.701907	0.570754
H	-2.598082	-0.383190	1.552783
H	-1.364507	-1.632504	1.849063
H	-0.247603	0.761141	1.944279
H	0.966203	-1.225279	1.280248
H	-0.043091	-2.639058	-0.259781
H	0.088460	-1.434515	-1.552219
H	-2.399175	-1.959463	-0.582244

TABLE S86: Cartesian coordinates in Angstroms (Å) for the M06-2X/TZ optimized 2,6-disubstituted norbornane −HB conformation with an PH_2 donor and OH acceptor group.

Atom	x	y	z
O	0.501868	2.033078	-0.036470
H	0.374134	2.572592	-0.819765
P	2.235522	-0.221105	-0.191229
H	2.639082	0.428610	0.998311
H	2.974265	-1.398816	0.101717
C	-1.718356	-0.662653	0.949693
C	-1.607474	-0.771619	-0.581621
C	-0.264278	-1.496697	-0.731578
C	0.591785	-0.889768	0.422892
C	-0.386482	0.089535	1.110037
C	-0.680896	1.313139	0.243011
C	-1.403292	0.700652	-0.987073
H	-0.783664	0.789531	-1.881801
H	-2.351222	1.202645	-1.177936
H	-1.372233	1.962926	0.789675
H	-2.579203	-0.080801	1.281476
H	-1.725412	-1.631899	1.447346
H	-0.088085	0.348273	2.123834
H	0.812889	-1.667242	1.151169
H	-0.383365	-2.572293	-0.600828
H	0.178212	-1.329014	-1.714591
H	-2.433957	-1.261291	-1.090982

TABLE S87: Cartesian coordinates in Angstroms (Å) for the df-MP2/TZ optimized 2,6-disubstituted norbornane −HB conformation with an PH_2 donor and OH acceptor group.

Atom	x	y	z
O	0.487596	2.012519	-0.028523
H	0.355874	2.533697	-0.826306
P	2.171128	-0.260961	-0.206397
H	2.625283	0.356711	0.980167
H	2.887151	-1.461308	0.042704
C	-1.771222	-0.648999	0.964773
C	-1.662200	-0.774016	-0.564196
C	-0.331432	-1.519078	-0.706193
C	0.532749	-0.907508	0.439566
C	-0.431116	0.087482	1.119768
C	-0.711144	1.304550	0.244748
C	-1.430644	0.688836	-0.983575
H	-0.801398	0.754947	-1.873025
H	-2.368307	1.204186	-1.189249
H	-1.402006	1.962274	0.781993
H	-2.624570	-0.052632	1.291137
H	-1.789908	-1.613843	1.471361
H	-0.130889	0.348316	2.132841
H	0.750641	-1.679417	1.176212
H	-0.463581	-2.591657	-0.561719
H	0.113070	-1.366028	-1.690985
H	-2.496494	-1.255219	-1.069933

TABLE S88: Cartesian coordinates in Angstroms (Å) for the df-MP2/aTZ optimized 2,6-disubstituted norbornane −HB conformation with an PH_2 donor and OH acceptor group.

Atom	x	y	z
O	0.496028	1.997451	-0.058099
H	0.331615	2.604598	-0.788061
P	2.183274	-0.276896	-0.193055
H	2.614795	0.362522	0.991796
H	2.893781	-1.473230	0.093374
C	-1.768300	-0.644919	0.971472
C	-1.671836	-0.767616	-0.559334
C	-0.335989	-1.502913	-0.718113
C	0.530486	-0.906350	0.433931
C	-0.426676	0.092907	1.116931
C	-0.711025	1.308833	0.241079
C	-1.456346	0.698321	-0.975790
H	-0.847451	0.776136	-1.878980
H	-2.402638	1.210448	-1.153510
H	-1.381652	1.980092	0.788398
H	-2.620669	-0.049133	1.304574
H	-1.779344	-1.610639	1.478484
H	-0.119286	0.355708	2.128353
H	0.735606	-1.685219	1.168595
H	-0.460994	-2.579448	-0.591806
H	0.105105	-1.328444	-1.701755
H	-2.508190	-1.254032	-1.058774

TABLE S89: Cartesian coordinates in Angstroms (Å) for the M06-2X/TZ optimized 2,6-disubstituted norbornane −HB anti conformation with an $PHCH_3$ donor and OH acceptor group.

Atom	x	y	z
O	-0.624737	2.163752	-0.208763
H	-0.355447	2.692235	0.547001
P	1.846120	0.570316	-0.240006
H	1.986902	1.340471	0.944504
C	3.293240	-0.557579	0.059874
H	3.327572	-0.951423	1.074725
H	3.223480	-1.392464	-0.638226
H	4.220702	-0.027540	-0.151576
C	-1.736855	-1.111386	0.977443
C	-1.636533	-1.184521	-0.556894
C	-0.133287	-1.428425	-0.747419
C	0.506606	-0.574076	0.390905
C	-0.719735	0.036079	1.102576
C	-1.427468	1.091555	0.241595
C	-1.943666	0.269030	-0.960572
H	-1.414305	0.571416	-1.864098
H	-3.008647	0.429626	-1.121067
H	-2.273233	1.486713	0.814052
H	-2.732226	-0.839687	1.331634
H	-1.414679	-2.026120	1.474614
H	-0.497816	0.378755	2.113213
H	0.984498	-1.240088	1.111252
H	0.111482	-2.483649	-0.624197
H	0.202505	-1.120866	-1.738869
H	-2.268192	-1.923181	-1.044327

TABLE S90: Cartesian coordinates in Angstroms (Å) for the df-MP2/TZ optimized 2,6-disubstituted norbornane −HB anti conformation with an $PHCH_3$ donor and OH acceptor group.

Atom	x	y	z
O	-0.618086	2.139462	-0.208334
H	-0.388757	2.680289	0.554740
P	1.834438	0.520733	-0.219255
H	2.016379	1.275911	0.967257
C	3.258428	-0.642966	0.045808
H	3.290858	-1.058543	1.051674
H	3.169201	-1.460732	-0.669019
H	4.195886	-0.128600	-0.159787
C	-1.758692	-1.122629	0.996968
C	-1.646038	-1.210361	-0.534497
C	-0.144841	-1.466009	-0.710115
C	0.489522	-0.604449	0.425585
C	-0.735982	0.018789	1.122167
C	-1.430009	1.068841	0.248826
C	-1.932965	0.241235	-0.953375
H	-1.384535	0.529951	-1.849669
H	-2.993334	0.411165	-1.133442
H	-2.281739	1.467365	0.809655
H	-2.755658	-0.840813	1.339835
H	-1.446020	-2.034953	1.505338
H	-0.520757	0.365644	2.133039
H	0.959380	-1.266380	1.156847
H	0.093603	-2.521645	-0.576124
H	0.200236	-1.165002	-1.700750
H	-2.280069	-1.948072	-1.021403

TABLE S91: Cartesian coordinates in Angstroms (Å) for the df-MP2/aTZ optimized 2,6-disubstituted norbornane −HB anti conformation with an $PHCH_3$ donor and OH acceptor group.

Atom	x	y	z
O	-0.607244	2.147701	-0.190893
H	-0.415468	2.709292	0.569041
P	1.827698	0.517403	-0.223611
H	2.032059	1.270879	0.961364
C	3.246702	-0.658699	0.014517
H	3.290397	-1.074028	1.020962
H	3.134821	-1.475107	-0.699852
H	4.184623	-0.150276	-0.207173
C	-1.766522	-1.117067	0.996461
C	-1.643066	-1.206069	-0.535114
C	-0.142376	-1.474058	-0.696496
C	0.488154	-0.604687	0.436327
C	-0.739204	0.020523	1.128440
C	-1.426101	1.072153	0.251503
C	-1.913879	0.248364	-0.959873
H	-1.346141	0.532791	-1.846709
H	-2.970528	0.425971	-1.158548
H	-2.283865	1.470875	0.803981
H	-2.765058	-0.830151	1.333217
H	-1.459529	-2.031432	1.506452
H	-0.527746	0.367711	2.140923
H	0.962823	-1.260590	1.171526
H	0.086883	-2.530786	-0.548802
H	0.214702	-1.184271	-1.687329
H	-2.279218	-1.940020	-1.027080

TABLE S92: Cartesian coordinates in Angstroms (Å) for the M06-2X/TZ optimized 2,6-disubstituted norbornane −HB gauche conformation with an $PHCH_3$ donor and OH acceptor group.

Atom	x	y	z
O	0.221982	2.006654	-0.531648
H	0.659018	2.598468	0.084612
P	1.836502	-0.491355	-0.656906
H	2.392578	-1.796407	-0.569450
C	2.855832	0.236253	0.712984
H	2.773445	-0.325812	1.642890
H	3.900825	0.267538	0.408997
H	2.528162	1.259693	0.889121
C	-1.849792	-0.454534	1.215102
C	-2.035760	-0.706837	-0.292152
C	-0.802690	-1.565104	-0.601384
C	0.295926	-0.934795	0.307138
C	-0.461068	0.189219	1.052241
C	-0.844388	1.357717	0.133818
C	-1.801156	0.696356	-0.882864
H	-1.322198	0.658856	-1.861452
H	-2.727068	1.261068	-0.980367
H	-1.393431	2.091182	0.733013
H	-2.589450	0.228711	1.634731
H	-1.831763	-1.371269	1.804071
H	0.034270	0.504100	1.970550
H	0.597612	-1.657460	1.066113
H	-0.971750	-2.608728	-0.335830
H	-0.541309	-1.528663	-1.660052
H	-2.977076	-1.162435	-0.589538

TABLE S93: Cartesian coordinates in Angstroms (Å) for the df-MP2/TZ optimized 2,6-disubstituted norbornane −HB gauche conformation with an $PHCH_3$ donor and OH acceptor group.

Atom	x	y	z
O	0.209175	2.007186	-0.503530
H	0.628238	2.596825	0.130900
P	1.805040	-0.501080	-0.623168
H	2.343056	-1.813348	-0.554713
C	2.849200	0.197747	0.741552
H	2.769043	-0.370319	1.667347
H	3.891308	0.213639	0.426483
H	2.543111	1.224789	0.929071
C	-1.876779	-0.449071	1.240531
C	-2.054874	-0.707953	-0.265220
C	-0.824418	-1.570768	-0.562765
C	0.270213	-0.937194	0.348461
C	-0.485937	0.189988	1.084418
C	-0.862693	1.353850	0.162184
C	-1.806043	0.688634	-0.861828
H	-1.312476	0.639647	-1.832290
H	-2.727480	1.257115	-0.977742
H	-1.420656	2.084615	0.756344
H	-2.616743	0.239164	1.652436
H	-1.864225	-1.363382	1.834102
H	0.002283	0.505294	2.006443
H	0.568527	-1.657533	1.112601
H	-0.995913	-2.613269	-0.293065
H	-0.556075	-1.536319	-1.620003
H	-2.996973	-1.160431	-0.566685

TABLE S94: Cartesian coordinates in Angstroms (Å) for the df-MP2/aTZ optimized 2,6-disubstituted norbornane −HB gauche conformation with an $PHCH_3$ donor and OH acceptor group.

Atom	x	y	z
O	0.207845	2.006404	-0.509288
H	0.609852	2.622654	0.112659
P	1.800938	-0.494822	-0.627329
H	2.338601	-1.808868	-0.566079
C	2.852663	0.200287	0.734008
H	2.773261	-0.374884	1.656456
H	3.893669	0.214887	0.412686
H	2.546177	1.226988	0.927093
C	-1.874781	-0.447025	1.247197
C	-2.053260	-0.715357	-0.257861
C	-0.821103	-1.578477	-0.549679
C	0.273644	-0.933787	0.354241
C	-0.484880	0.194727	1.086168
C	-0.866746	1.352005	0.157554
C	-1.805895	0.678003	-0.865157
H	-1.308167	0.622030	-1.834230
H	-2.728860	1.244348	-0.987534
H	-1.426047	2.086829	0.746379
H	-2.616443	0.242511	1.656187
H	-1.858071	-1.359196	1.845526
H	0.004866	0.517988	2.005710
H	0.581644	-1.648327	1.121587
H	-0.990526	-2.619388	-0.269275
H	-0.552850	-1.552378	-1.608154
H	-2.995563	-1.171722	-0.556375

TABLE S95: Cartesian coordinates in Angstroms (Å) for the M06-2X/TZ optimized 2,6-disubstituted norbornane −HB conformation with an $P(CH_3)_2$ donor and OH acceptor group.

Atom	x	y	z
O	-0.626644	2.062324	-0.723511
H	-0.380922	2.791509	-0.149743
P	1.669493	0.132274	-0.515656
C	2.344885	1.242102	0.803461
H	2.391122	0.738079	1.771200
H	3.348706	1.562050	0.525535
H	1.722653	2.131093	0.895775
C	2.850199	-1.279061	-0.286912
H	2.969364	-1.551447	0.763841
H	2.483944	-2.146190	-0.837190
H	3.823147	-1.010972	-0.697718
C	-1.985364	-0.720944	1.227914
C	-2.000560	-1.161222	-0.247194
C	-0.554217	-1.639726	-0.426952
C	0.256832	-0.626134	0.436732
C	-0.838043	0.286954	1.033527
C	-1.491532	1.194978	-0.017872
C	-2.153560	0.184679	-0.980796
H	-1.630149	0.197239	-1.936931
H	-3.197329	0.436296	-1.162400
H	-2.264392	1.786710	0.483655
H	-2.915987	-0.251211	1.549384
H	-1.734503	-1.529621	1.914052
H	-0.511399	0.821656	1.925339
H	0.713654	-1.153151	1.279353
H	-0.426268	-2.656593	-0.055074
H	-0.253499	-1.627563	-1.476195
H	-2.748029	-1.902665	-0.518773

TABLE S96: Cartesian coordinates in Angstroms (Å) for the df-MP2/TZ optimized 2,6-disubstituted norbornane −HB conformation with an $P(CH_3)_2$ donor and OH acceptor group.

Atom	x	y	z
O	-0.623873	2.051735	-0.699309
H	-0.400827	2.782146	-0.113925
P	1.662552	0.108415	-0.487981
C	2.362442	1.198225	0.832445
H	2.412657	0.684909	1.794747
H	3.367695	1.508188	0.548129
H	1.754267	2.094398	0.938841
C	2.826398	-1.316208	-0.269546
H	2.936381	-1.598742	0.779369
H	2.457496	-2.174970	-0.829957
H	3.804727	-1.054117	-0.671347
C	-1.987612	-0.731988	1.254679
C	-2.001242	-1.173889	-0.218577
C	-0.557371	-1.655090	-0.395696
C	0.253153	-0.641590	0.469083
C	-0.838499	0.273225	1.063001
C	-1.489118	1.179625	0.012523
C	-2.143328	0.170200	-0.953972
H	-1.608701	0.178595	-1.903642
H	-3.184153	0.425292	-1.147249
H	-2.265881	1.766366	0.513764
H	-2.918106	-0.259186	1.573523
H	-1.738956	-1.540359	1.942631
H	-0.514281	0.805448	1.957211
H	0.709109	-1.169436	1.313586
H	-0.430183	-2.672399	-0.023949
H	-0.255446	-1.638852	-1.444715
H	-2.751128	-1.913199	-0.491387

TABLE S97: Cartesian coordinates in Angstroms (Å) for the df-MP2/aTZ optimized 2,6-disubstituted norbornane −HB conformation with an $P(CH_3)_2$ donor and OH acceptor group.

Atom	x	y	z
O	-0.617536	2.053168	-0.701117
H	-0.427831	2.804922	-0.129112
P	1.657426	0.111655	-0.484225
C	2.366004	1.197884	0.834742
H	2.421159	0.675108	1.792702
H	3.369944	1.508445	0.543762
H	1.755390	2.092291	0.949593
C	2.817005	-1.317831	-0.273012
H	2.927527	-1.599957	0.776913
H	2.440469	-2.174107	-0.833619
H	3.794893	-1.055974	-0.678129
C	-1.994954	-0.728067	1.250969
C	-1.995475	-1.178467	-0.220847
C	-0.549834	-1.661294	-0.380235
C	0.252452	-0.636540	0.479640
C	-0.845106	0.278174	1.063094
C	-1.490322	1.177313	0.002999
C	-2.129049	0.161501	-0.967731
H	-1.578776	0.164302	-1.909618
H	-3.168037	0.414437	-1.177899
H	-2.274024	1.765412	0.492814
H	-2.929205	-0.254163	1.560029
H	-1.749108	-1.533426	1.944808
H	-0.527340	0.817157	1.956707
H	0.712860	-1.154565	1.329341
H	-0.425888	-2.674634	0.005745
H	-0.236984	-1.656380	-1.427216
H	-2.743625	-1.919952	-0.496461
